# The Shape of Things to Come: α‑Helical Membrane Protein Folding on the Ribosome

**DOI:** 10.1021/acs.chemrev.6c00236

**Published:** 2026-05-18

**Authors:** Edward Lambden, Benjamin Russell Lewis, Zadie L. R. Baker, Marvin V. Dilworth, Erin C. Johnston, Juan Palacios-Ortega, Kaylee Patel, Colin P. Pilkington, Heather E. Findlay, Paula J. Booth, Grant A. Pellowe

**Affiliations:** † Aston Institute for Membrane Excellence, Aston University, Birmingham, B4 7ET, U.K.; ‡ Department of Chemistry, 4616 King’s College London, Britannia House, 7 Trinity Street, London, SE1 1DB, U.K.; § The Francis Crick Institute, 1 Midland Road, London, NW1 1AT, U.K.; ∥ Centre for the Physical Science of Life, Strand Building, Strand Campus, Strand, London, WC2R 2LS, U.K.

## Abstract

Understanding how membrane proteins insert into and fold within cell membranes is critical for explaining the molecular basis of many diseases. It also underpins advances in biotechnology, including the development of therapies for protein misfolding disorders and improved methods for producing membrane proteins at high yield. In cells, nearly all α-helical membrane proteins are synthesized and inserted cotranslationally, folding sequentially as they emerge from the ribosome. This process occurs under spatial constraints imposed by the translational machinery and in membranes with complex physicochemical properties. These processes are vastly different from classical *in vitro* refolding studies of full-length purified proteins, highlighting a critical need to alter our experimental approach to understand *de novo* membrane protein folding. The mechanisms driving membrane protein folding remain elusive, largely due to the limited availability of approaches that can probe these processes both in real-time and in their native context. Here, we discuss recent progress in uncovering how membrane proteins fold during synthesis and insertion, and highlight how established and emerging biophysical and structural tools are beginning to resolve cotranslational events with greater mechanistic detail than has been previously possible. Together, these advances are reshaping our understanding of membrane protein biogenesis far beyond traditional refolding models.

## Graphical abstract

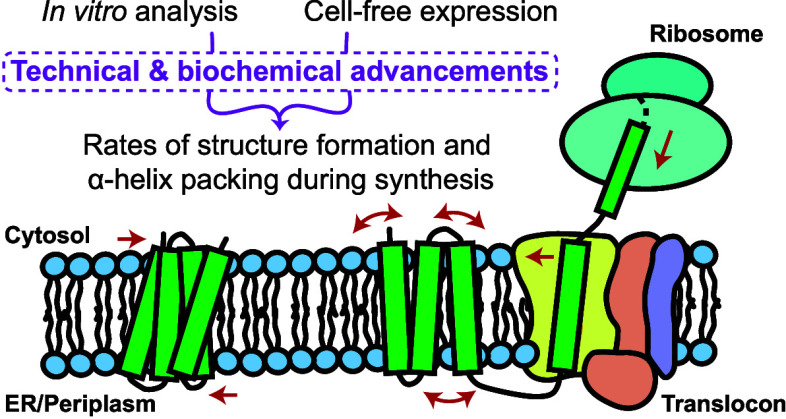

## 1 Introduction

Membrane proteins constitute roughly one-quarter of all proteomes and represent over half of current drug targets, yet how they fold remains far less understood than their soluble counterparts. For α-helical integral membrane proteins, folding is not a post-translational event: it begins cotranslationally, as nascent chains emerge from the ribosome, engage the translocon, and partition into the lipid bilayer. The coupling of translation to folding imposes unique constraints on the nascent chain: from vectorial (N- to C-terminus) synthesis, sequential exposure of hydrophobic transmembrane segments, and a crowded interface populated by chaperones and quality-control machinery. These paradigms distinguish cotranslational membrane protein biogenesis from classical refolding scenarios of isolated proteins, which are often recombinantly produced. Disruption of the cotranslational process underlies a growing list of human diseases and has motivated interest in developing pharmacological chaperones that rescue misfolded variants.^1^

Early conceptual models of membrane protein folding have provided important frameworks for understanding membrane protein stability, most notably the two-stage hypothesis,^2^ which proposed that transmembrane helices form independently before assembling laterally, together with thermodynamic models describing partitioning between aqueous, interfacial, and hydrocarbon environments. However, these models are largely based on equilibrium refolding of fully synthesized, isolated proteins. As a result, they do not capture the complexity of cotranslational folding, where protein synthesis, translocation, and membrane insertion occur simultaneously. In this context, several kinetic processes, including helix insertion, helix–helix packing, and nascent chain elongation, proceed at the same time and must be tightly coordinated. Imbalances among these rates can lead to misfolding, aggregation, or kinetically trapped intermediates.

At present, we lack direct measurements of these individual rates, leaving a significant gap in our mechanistic understanding of cotranslational membrane-protein folding. A comprehensive understanding will require quantitative measurements of both the thermodynamic stability of individual transmembrane helices and the kinetic parameters that govern their insertion and assembly during the synthesis.

In this review, we focus on the cotranslational folding of α-helical integral membrane proteins at the ribosome–translocon–bilayer interface (Figure 1). In Section 2, we discuss the mechanisms proposed to link nascent chain emergence from the ribosome to membrane insertion and early folding. We draw on work from both bacterial systems, including SecYEG and YidC, and eukaryotic systems, including Sec61 and the endoplasmic reticulum (ER) membrane complex (EMC), to highlight conserved and divergent features of translocon-assisted integration. In Section 3, we examine how cotranslational folding differs from equilibrium refolding models. We discuss the kinetic constraints, transient insertion intermediates, and contributions from the ribosome and translocon that shape folding pathways during ongoing synthesis. In Section 4, we review recent methodological advances that enable quantitative investigation of these processes. We discuss how cell-free translation systems^3–5^ allow membrane protein biogenesis to be reconstituted in defined environments and monitored in real-time using surface-enhanced infrared absorption spectroscopy (SEIRAS).^6–8^ We note arrest-peptide force profiling (FPA)^9,10^ to measure insertion energetics, as well as single-molecule fluorescence^11,12^ and complementary biochemical assays to probe folding mechanisms.^13–21^ We also describe how ribosome-bound nascent chain complexes (RNCs) can be stalled and isolated for analysis.^22–25^ In Section 5, we discuss how molecular simulations^26,27^ may be applicative to membrane protein cotranslational folding to provide greater insight into the unknown. Finally, in Section 6, we discuss how emerging mechanistic models of cotranslational membrane protein folding inform our understanding of disease and its molecular origins.

Despite the recent methodological and biophysical advances applied to explore cotranslational folding, several key challenges remain. A key issue is that the majority of work to date has focused on the mechanism of translocon-mediated insertion, leaving the subsequent process of cotranslational folding within the lipid bilayer largely unexplored (Figure 1). Consequently, fundamental questions about when and how structure forms remain unanswered. **1)** We lack predictive rules linking protein sequence and lipid environment to cotranslational folding pathways, including how helical structure forms during synthesis and insertion. For instance, it is unknown whether α-helical structure forms cooperatively during synthesis and insertion, or even if helicity is the default state, as the structure of the nascent chain within the ribosome tunnel and translocon may be more dynamic than current models suggest. There are currently no reports directly observing this cotranslational process in real-time in the native membrane. **2)** Intermediate states are transient and difficult to capture at physiological time scales, making it hard to define folding trajectories and quantify their kinetics as the nascent chain moves from the ribosome, through the translocon, and into the bilayer. **3)** Integrating translocon gating with helix–helix interaction energetics and the lateral pressure profile of the bilayer remains a major unresolved question. A framework that connects these physical factors to structure formation and kinetic barriers is still missing. **4)** Translating mechanistic insight into clinical understanding of variant pathogenicity is only beginning, and it requires clearer links between altered folding kinetics, misfolded structural states, and cellular dysfunction. We therefore highlight emerging methodologies in which convergent, integrative approaches can bridge these fundamental knowledge gaps and advance our understanding of integral membrane protein biogenesis.

## 2 Background

### 2.1 Fundamentals of Membrane Protein Folding

Integral α-helical membrane proteins must be targeted to the membrane for folding, as they are hydrophobic and form transmembrane domains (TMDs). If released into the cytosol, these domains misfold and aggregate. Historically, membrane protein folding was described by the two-stage model proposed by Popot and Engelman in 1990,^2^ later expanded to three stages in 2003.^28^ In this framework, TMDs first insert into the lipid bilayer as they emerge from the ribosome ­(Figure 2a), driven primarily by the hydrophobic effect, as the apolar residues of TMDs preferentially associate with the lipid bilayer. Subsequently these helices interact with each other to form higher order structures followed by further folding events,^28^ including folding of extramembrane groups postinsertion, creation of binding surfaces and other domain association events.^29–32^

**Figure 1:**
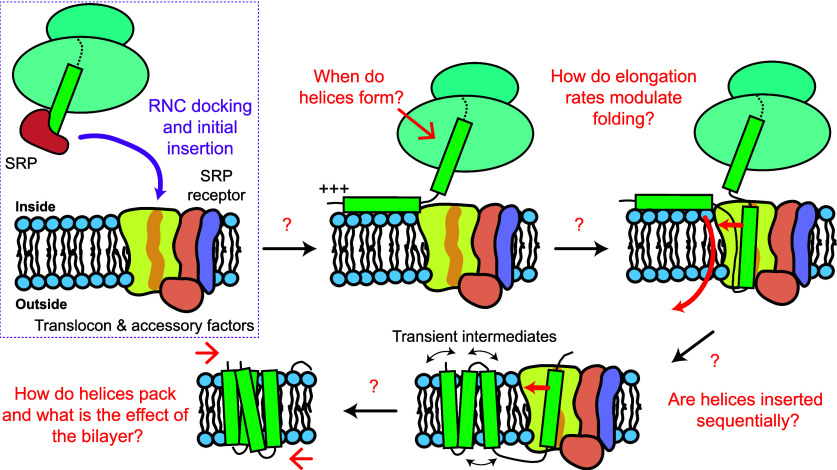
**Cotranslational membrane protein folding and outstanding questions.** Most studies of membrane protein biogenesis have focused on cotranslational insertion (purple box), primarily using in vitro translation and biochemical approaches. However, several key aspects of the overall process remain unresolved. It is unclear when transmembrane helices first form, either within the ribosome exit tunnel or after reaching the membrane. The mechanisms by which helices pass through the translocon and partition into the lipid bilayer are also not fully understood. It also remains uncertain whether helices insert sequentially and how the surrounding lipid environment influences helix packing within the membrane. Insertion and folding are further modulated by the ribosome elongation rate. Because time-resolved methods that track structural acquisition during translation are limited, and transient ribosome-bound intermediates are difficult to capture, many fundamental questions in membrane protein folding remain unanswered.

In the cell, transmembrane helical proteins can adopt two key orientations upon membrane insertion (Figure 2b). In bacteria, the N-out (type I) topology corresponds to the N-terminus of the first TM helix facing the cytosolic side, whereas the N-in (type II) topology corresponds to the N-terminus oriented toward the extracytoplasmic side of the membrane,^33^ with the N-out topology generally favored. The final orientation is largely dictated by the distribution of charged residues near the N-terminus of each transmembrane helix, with positively charged residues preferentially found on the cytosolic side (Figure 2c). This has been named the “positive inside rule”.^34^ For proteins adopting the N-in topology, it is proposed that the TMDs may invert during synthesis or insertion; however, the exact mechanism remains unclear.^35^ How these TMDs orientate themselves is crucial for the correct biological function of the protein. In cells, the insertion process is generally mediated by the translocon or other membrane insertase proteins, with the translocon also functioning as a channel for the passage of secreted proteins through the bilayer. In addition to the impact of charged residues,^36^ strongly hydrophobic segments within membrane protein sequences, also called stop/transfer sequences, or signal-anchor sequences control what remains in the membrane (as TMDs) and what ends up in the cytosol. The stretch of hydrophobicity will stop the transport of the protein across the membrane and prolong its time interacting with the translocon, promoting that section to become embedded, instead of fully secreted.^37^

The lipid bilayer itself is an important component of the membrane protein folding process, not merely a static spectator but an active regulator of integral membrane protein stability (Figure 2c).^38–40^ Native biological membranes comprise diverse lipid species with dynamic compositions; bacteria tune lipid content in response to temperature, pH, and pressure,^41,42^ while that of humans is affected by diet, stress disorders,^43^ or disease.^44^ The lipid bilayer and membrane as a whole are important in describing the free energy landscape for folding. Moreover, while folding, proteins are susceptible to forces applied by the lipid membrane. Insights into the influence of the lipid bilayer on membrane protein biogenesis deepens our understanding of cellular organization and opens therapeutic avenues for diseases of membrane protein misfolding. For eukaryotes in particular, the precise folding and fold-maintenance of α-helical membrane proteins is likely to be reliant on the different membranes they experience during trafficking (plasma membrane, mitochondria, endoplasmic reticulum).

While significant and nascent work has been performed on the cotranslational folding of individual proteins, a comparatively underexplored question surrounds the mechanisms of quaternary membrane protein assembly. Some complexes have been shown to assemble post-translationally, in a process mediated by molecular chaperones.^45^ However, for other complexes and multimers, the interaction between components is more complicated and how their assembly occurs is more difficult to understand. For example, AcrB has a crucial loop for stabilizing its homotrimeric structure: each monomer’s loop is integrated into its neighboring subunit,^46^ with its structure intact during assembly.^47^ Whether this interaction is formed cotranslationally, with monomers building on to each other, or post-translationally, where individual subunits are held in proximity for the final assembly, is unknown. Given the critical role of efflux pumps such as AcrAB–TolC in multidrugresistant bacteria, understanding how their structure forms is crucial for developing effective antimicrobial drugs.^48,49^ Although these types of folding processes are important and the possibility that quaternary structures can arise cotranslationally is fascinating, there is, as yet, little direct evidence of this for membrane proteins. Therefore, this review focuses on the more widely explored and studied monomeric membrane proteins.

Another major class of integral membrane proteins are those with a β-barrel structure. These are generally found in the outer membranes of Gram-negative bacteria, mitochondria and chloroplasts. They fold post-translationally rather than cotranslationally, in association with periplasmic chaperone proteins and the β-barrel assembly machinery (BAM) complex, the process of which has been reviewed elsewhere.^50,51^

### 2.2 Cotranslational Membrane Protein Folding in Bacteria

*In vivo*, the majority of bacterial membrane proteins are inserted cotranslationally ­(Figure 3) with the assistance of a protein-conducting channel, known as the translocon.^52^ This process begins as the nascent polypeptide emerges from the ribosome. Most integral membrane proteins contain an N-terminal signal sequence that is recognized by a bacterial signal recognition particle (SRP). In Gram-negative bacteria, the SRP consists of the protein Ffh and a 4.5S RNA component,^53^ whereas in Gram-positive bacteria it contains a larger 6S RNA.^54^ SRP is then recognized by its receptor, FtsY, which interacts with the bacterial translocon and the surrounding phospholipids via its disordered A domain. This complex hands the nascent chain over from the SRP to the translocon to facilitate the protein insertion into the membrane.^55^

**Figure 2:**
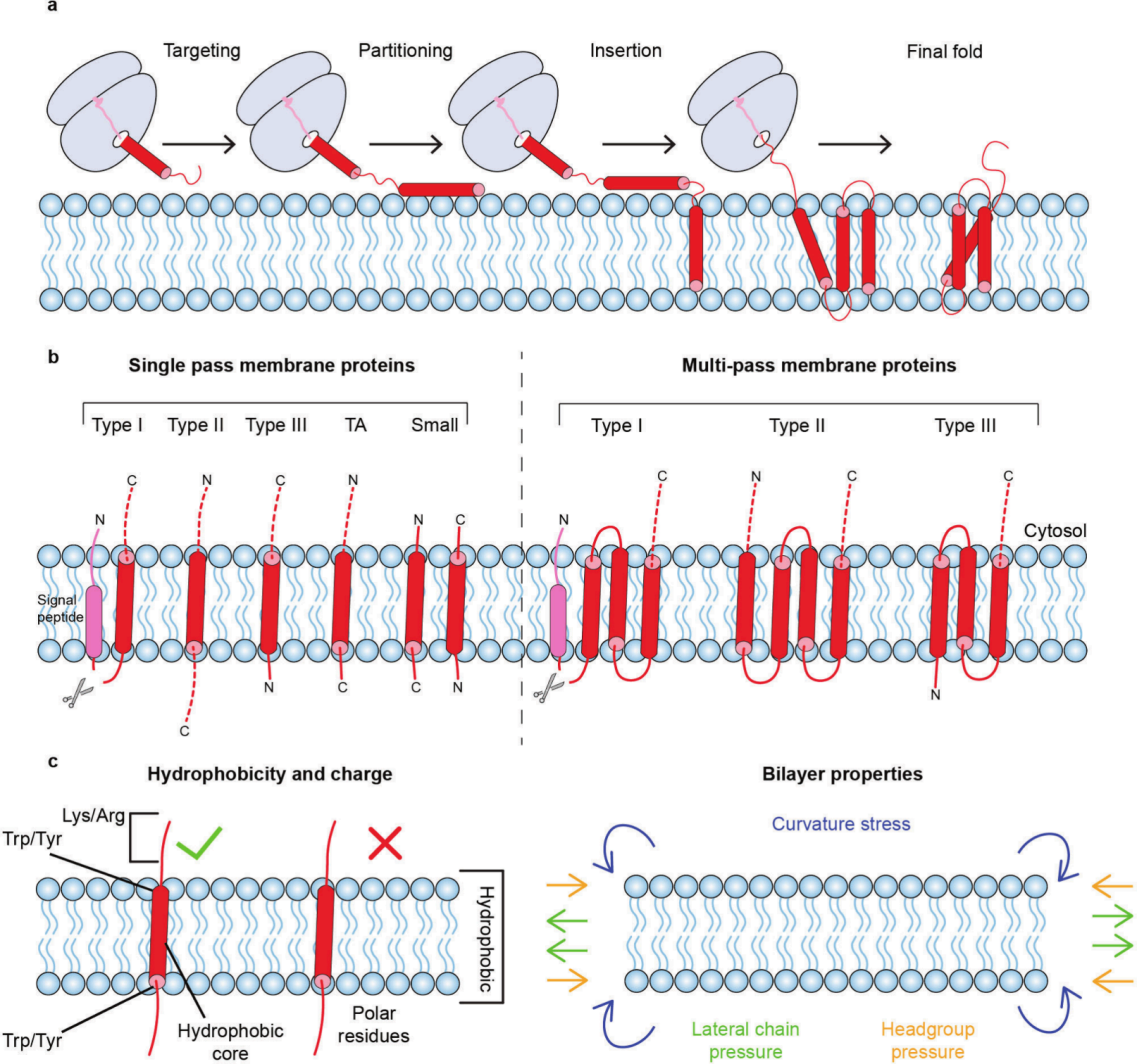
**Introducing integral α-helical membrane protein folding. a.** The steps involved in the spontaneous (translocon void) folding of α-helical membrane proteins. TMDs are targeted to the membrane and undergo TM interfacial headgroup partitioning and TMD insertion into the bilayer, before reaching the final fold. This can occur with or without the presence of the translocon. **b.** Different classes of integral membrane protein and their topologies. Dotted red line indicates continued protein sequence. TA; Tail anchored. **c.** Forces driving protein folding in the bilayer. The physiochemical make up of a TMD can promote or hinder insertion into the bilayer. Bilayer properties detailed, with curvature stress, headgroup pressure and lateral chain pressure being prominent forces in the bilayer.

Structural studies have elucidated the architecture of the bacterial core translocon, which has been determined to be a ternary complex, consisting of SecY, SecE, and SecG (collectively known as SecYEG). The primary component of the bacterial core translocon is SecY, comprising ten TMDs that are arranged in a pseudosymmetrical fashion, with the TMs 1–5 and 6–10 forming a central pore through which secreted proteins can pass into the extracytoplasmic side of the membrane.^11^ For membrane proteins, the current dominant hypothesis is that the two halves of SecY undergo lateral movements upon ribosome-nascent chain binding, opening a lateral gate that facilitates membrane insertion.^56^ SecE stabilizes the SecY complex by maintaining its structural integrity, while SecG is positioned more peripherally. Although SecG is not essential for translocation, it enhances the efficiency of translocon function. During insertion through the SecYEG translocon, recruitment of the RNC brings the ribosome into direct contact with the cytoplasmic loops of the translocon. This interaction forms a sealed and protected environment around the cytosolic vestibule of the channel. This arrangement both guides the nascent chain into the translocon pore and shields it from exposure to the cytosol during insertion.^57^

The SecYEG translocon is not the only mechanism by which membrane proteins can enter their native environments: some membrane proteins, especially small proteins that span the membrane only once (termed single-pass proteins), are proposed to be inserted into the membrane by YidC in a Sec-independent manner. There are two key structural motifs of YidC which could play a role in SecYEG independent insertion: the hydrophilic groove, which is responsible for substrate binding and handles hydrophilic regions, and the “greasy slide”, which is responsible for allowing lateral movement of the hydrophobic regions into the membrane.,^58,59^

Large proteins with multiple membrane-spanning segments (multipass proteins) or multiple domains may also be inserted into the membrane by YidC in a Sec-dependent manner via the holotranslocon (HTL) composed of SecYEG-SecDF-YajC-YidC. SecDF and YajC are accessory proteins that associate with YidC. While YajC is thought to play a role in helix insertion, SecD and F are primarily involved in periplasmic secretion.^60,61^ The HTL forms a lipid cavity in the bilayer to provide a protected environment for membrane-protein insertion, folding, and controlled release into the inner membrane. In this model of insertion, the membrane-protein chaperone YidC binds successive TMs exiting from the SecY lateral gate and an opening for the release of TM-bundles could then be achieved by YidC flexibly moving away from SecYEG.^61,62^ The HTL is a large and dynamic structure with many components and its surrounding lipid composition, with cardiolipin concentration in particular playing a role in stabilizing the complex.^63^ The lipid composition can therefore affect the folding of a nascent chain directly through the impact of the lipid environment on the nascent chain or indirectly through the impact of the lipid environment on parts of the machinery required for insertion and folding.

### 2.3 Cotranslational Membrane Protein Folding in Eukaryotes

In mammalian cells, the biogenesis of membrane proteins, except for those destined for mitochondria or peroxisomes, begins in the endoplasmic reticulum (ER). From the ER, proteins are trafficked through the Golgi apparatus to reach their correct destinations such as the plasma membrane. There have been many extensive recent reviews on mechanisms of multipass membrane protein insertion at the ER.^64–67^

While the overall mechanism of biogenesis is similar to that in bacteria, mammalian systems have additional complexity and evolutionary divergence from the HTL model described above ­(Figure 4).

**Figure 3:**
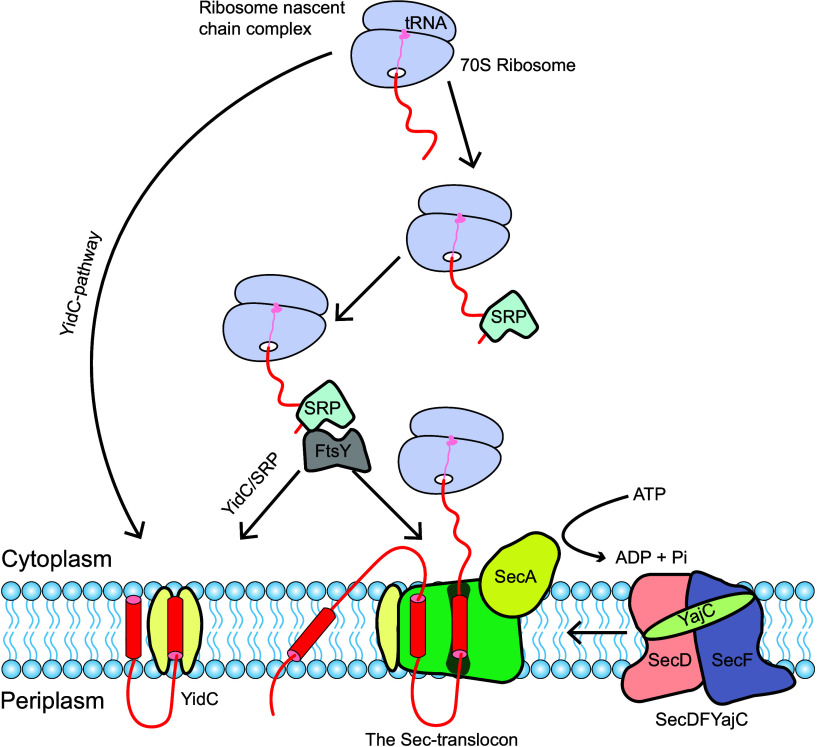
**Biogenesis of inner membrane protein in bacteria.** RNCs are targeted to the bacterial inner membrane by the signal recognition pathway (SRP and FtsY receptor). RNCs dock at SecYEG to facilitate the insertion of TMs into the bilayer. YidC insertase can assist the translocon or function independently via the SRP pathway or YidC pathway, respectively. SecDF-YajC can assist the translocon with membrane protein insertion or secretion.

The first TMD or a signal sequence encoded into the nascent protein decides the destination of the protein - the ER, mitochondria, or peroxisome - and is dependent on its charge and hydrophobicity. These informative selections are recognized in early biogenesis by the SRP, which delivers and docks the RNC to the translocon at the desired membrane.^65^

Upstream of the SRP is the nascent polypeptide associated complex (NAC), a heterodimer of NACα and NACβ (BTF3b in humans). NAC associates with ribosomes primarily through a charged motif in the unstructured N-terminal tail of NACβ. NAC triages the nascent chain by competition with SRP to prevent mistargeting of cytosolic or mitochondria bound proteins to the ER.^69^ Should an ER signal sequence be detected by the globular β-domain of NAC, which contains a hydrophobic cavity, a structural rearrangement of the complex occurs. This rearrangement detaches the globular domain from the exit tunnel, while bringing SRP into the vicinity of the nascent chain for delivery and subsequent handover of the RNC to the ER using its flexible N and C termini and ubiquitin-associated (UBA) domain.^70^ Ribosome profiling experiments suggested that approximately 30% of the ER proteome interacted with NAC and characterized this interaction in relation to its position along the reading frame of these ER-destined RNCs. The majority show NAC binding early in a protein sequence, often while the nascent chain is still in the ribosome, confirming that NAC largely acts as an initial triage for ER destined proteins.^71^ Although a further 15% of the NAC binding events were found to occur at codons later in the sequences, the significance of this for chaperoning, targeting or folding is as yet unclear.

RNCs targeted to the ER are introduced by the SRP to the Sec61 translocon, which forms a pore within the ER membrane where the signal sequence is inserted.^72^ As with SecYEG, the Sec61 translocon is used for both secretory and membrane proteins. Secretory proteins will fully cross the membrane through the Sec61 channel, as they do not have a high hydrophobic residue content. The insertion of membrane proteins into the membrane, after the Sec61 translocon, requires support from accessory proteins to ensure they adopt the correct orientation.^56^

The Sec61 translocon is made up of 3 subunits, Sec61α, Sec61β and Sec61γ. The Sec61α subunit consists of the central pore, through which the nascent chain is passed, and the lateral gate which is proposed to mediate access to the lipid bilayer. This first subunit is formed from 10 transmembrane helices that can be split into two halves, TM1–5 and TM6–10. The lateral gate in Sec61α interacts with the signal sequence on the nascent chain when the RNC complex binds via the SRP receptor. This causes the conformational change that opens the translocon at the lateral gate.^73^ There is a lack of research on the Sec61β subunit so its function is not fully elucidated; however, it is suspected that this small tail anchored accessory protein could aid in efficient ribosome docking and translocon stability during the conformational change. The final subunit, Sec61γ, appears to be an essential part of the Sec61 translocon complex. Its structure adjoins the two halves of Sec61α, TM1–5 and TM 6–10, and stabilizes the complex by acting as a clamp.^74^ Additionally, RAMP4 (and SERP1 in humans) often copurifies with Sec61 and appears to stabilize and widen the open conformation and thus the hydrophilic pore of the Sec61 lateral gate while retaining its tether to the ribosome.^73^

A variety of different larger protein complexes containing or interacting with RNC-Sec61 can be formed, depending on factors such as nascent chain sequence, post-translational modification, and organelle targeting. For example, the ER membrane protein complex (EMC), a 9-protein complex, has a wide range of functions across membrane protein biogenesis.^75^ One subunit; EMC3, is part of the OxaI insertase family (see YidC). Membrane proteins that require EMC tend to have TMDs of low hydrophobicity or utilize its role as a tail-anchored insertase post-translationally.^76^

The multipass translocon (MPT) is a large complex composed of RNC:Sec61 plus three obligate heterocomplexes of the GET- and EMC-like (GEL), protein associated with translocon (PAT) and back of Sec61 (BOS) complexes.^68^ MPT-mediated insertion does not appear to rely on the Sec61 lateral gate^77^ with the subcomplexes suggested to have a role in the assembly of multispanning membrane proteins with more polar regions (PAT), forming a lipid filled pore for multipass membrane protein folding and interacting with the EMC.^78^ There is no single insertase that works universally for client integration.^79^

**Figure 4:**
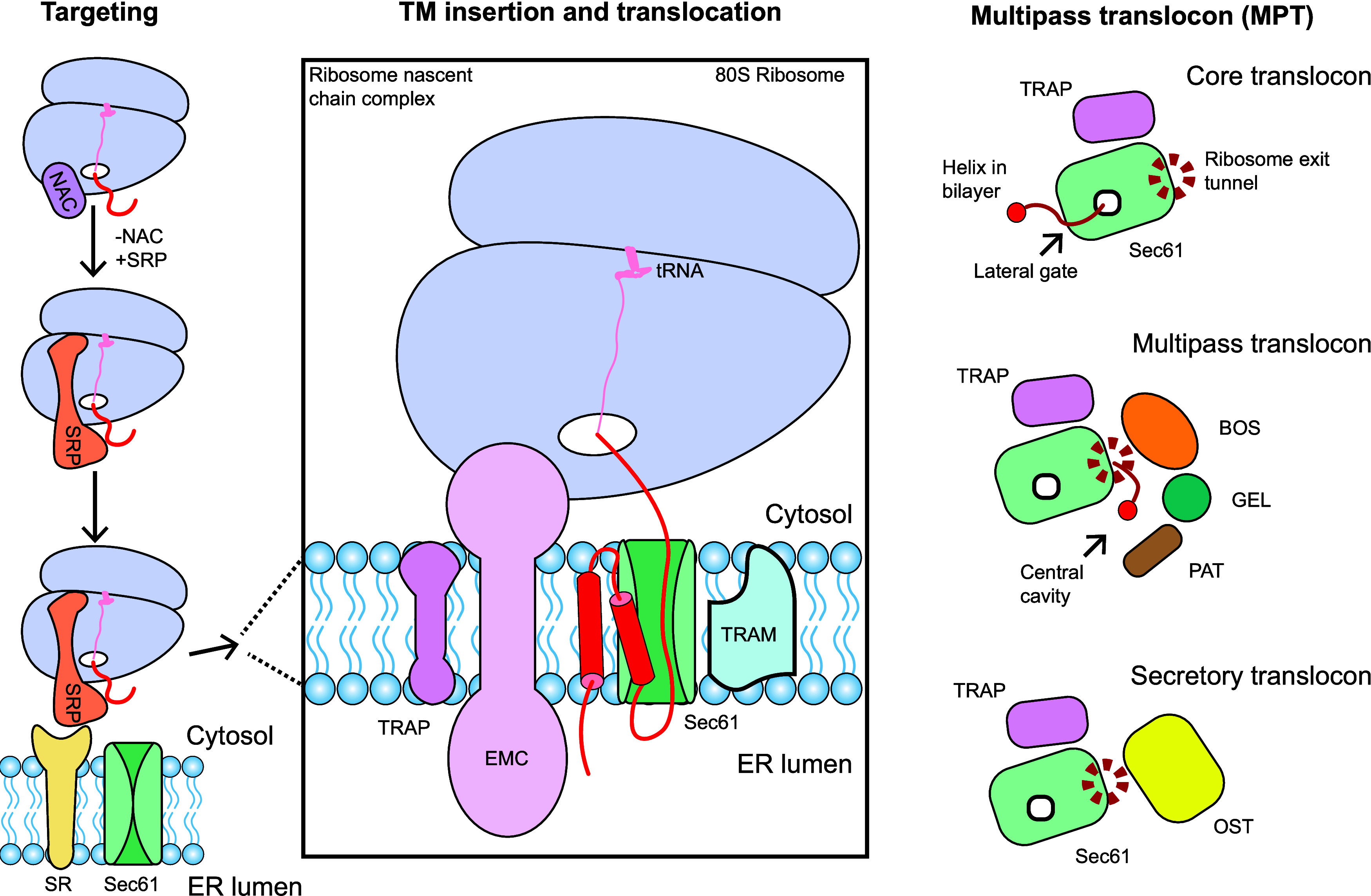
**Simplified view of biogenesis of inner membrane protein in eukaryotes.** NAC initially binds the RNC until a hydrophobic region or signal peptide emerges, where NAC is replaced by SRP. RNCs are targeted to the ER by SRP-SR, which hands over the nascent chain to the Sec61 translocon. The Sec61 works with EMC and other factors, including BOS, GEL and PAT which form the MPT. OST also associates with Sec61 to form the secretory translocon. MPT top-down views adapted from ref:^68^ Sundaram, A.; Yamsek, M.; Zhong, F.; Hooda, Y.; Hegde, R. S.; Keenan, R. J. Nature, 2022, 375, 839–844. Licensed under CC-BY 4.0. Copyright © 2022 Springer Nature.

Translocating Chain-Associating Membrane protein (TRAM) is another major accessory complex for the MPT which purifies at near 1:1 stoichiometry with Sec61:RNC complexes. TRAM’s main function is to determine the final topology and location of a membrane protein within a cell.^74^ TRAM translocates proteins with specific signal sequences postmigration through the Sec61 complex. These signal sequences that indicate a TRAM requirement tend to have shorter positively charged N regions, or altered transmembrane domain lengths, compared to proteins that do not require TRAM.

The translocon-associated protein complex (TRAP) is also associated with the MPT and guides the initial insertion of signal sequences specifically for secretory and membrane proteins. Both TRAM and TRAP work in conjunction with Sec61. The TRAP complex is only required for nascent proteins with certain signal sequences.^80^ TRAP is also suspected to play a role in the initial N-glycosylation of nascent proteins; this occurs during its interaction with oligosaccharyl transferase (OST).^81^ Also present at the MPT is the signal peptidase complex (SPC); this mediates, alongside OST, the cleavage of signal sequences resulting in Type I N_exo_ proteins.

## 3 *In Vitro* Studies of Folding

To fully unpick the mechanisms that promote correct *de novo* folding, it is helpful to understand the fundamental environment into which a membrane protein folds. Much of the original work has been carried out using *in vitro* methodologies to understand the global and chemical properties of the lipid environment, as well as the hydrophobic nature of nascent membrane proteins. Most of these studies have employed ‘bulk’ refolding approaches using purified proteins in various membrane mimetics, each of which imparts different levels of biological complexity. These complexities are discussed in this section.

Biological membranes are composed of many thousands of different types of lipid molecules, with a high degree of diversity found between different bacterial species, between prokaryotic and eukaryotic membranes, and even between different cells, organelles, and across the cell cycle.^82,83^ Cholesterol, in particular, is found at varying concentrations in different eukaryotic membranes and alters bilayer packing, fluidity, and bilayer thickness in a temperature-dependent manner.^84^ Membrane lipid composition plays a central role in shaping how membrane proteins insert and fold, not only in the ER but also in bacterial membranes. *In vitro* folding systems have shown that the first lipids encountered by a nascent polypeptide can influence TMD orientation, insertion efficiency, and early folding events by modulating bilayer properties, such as lateral pressure, surface charge, thickness, and fluidity. For example, membranes enriched in negatively charged lipids can promote transmembrane domain insertion by facilitating helix partitioning into the bilayer, whereas lipids that increase lateral chain pressure may hinder insertion while stabilizing proteins once embedded.^85^ Changes in cholesterol in eukaryotic membranes can reshape the energetics of helix insertion and compaction in the membrane.^86^ While in this review we largely focus on these overall bilayer properties, it should be noted that some proteins also require a specific lipid interaction to achieve the correct fold or function.^87–89^

Because bacterial and eukaryotic membranes (ER, mitochondria, peroxisomes, and the plasma membrane) differ substantially in lipid composition and physiochemical properties, the environment in which a membrane protein folds can vary drastically across organisms, and this fold must be maintained during trafficking across compartments.^1^ These differences therefore highlight the importance of *in vitro* refolding studies, where lipid composition can be precisely controlled to dissect the physical principles governing membrane protein folding, especially as measuring these parameters directly during *in vivo* folding has yet to be achieved.

### 3.1 Membrane Biophysics and Membrane Proteins

In the field of membrane protein biochemistry, micellar assemblies have been widely used as solubilizing agents, to maintain higher-order protein structures for purification and structural analysis by shielding hydrophobic regions from an aqueous environment.^90–94^ However, a greater appreciation for the intricacies of membrane biophysics has paved the way for a wider variety of amphiphilic compounds and lipid bilayer mimetics that can more closely reproduce the native lipid environment of a given membrane protein.^91,95,96^ (Figure 5a) This is particularly useful in the case of cotranslational folding as, while a simple detergent micelle may sufficiently stabilize a fully folded membrane protein for structural elucidation, folding kinetics and mechanistic studies are more elusive, requiring a delicate balance of physical properties such as lateral pressures, charge, and curvature.^90–96^

**Figure 5:**
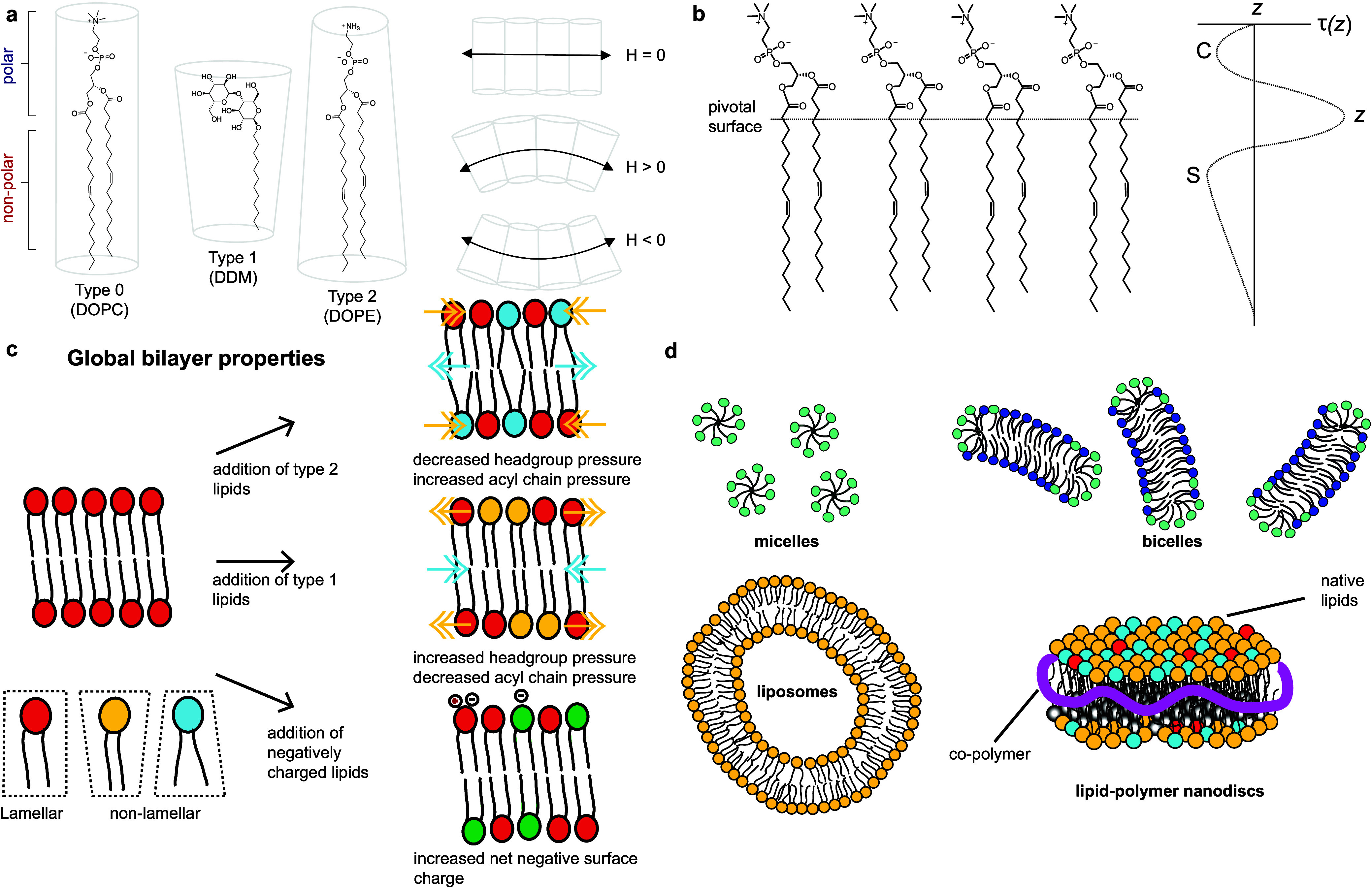
**Physiochemical properties of lipids and common mimetics. a.** Graphical illustration of amphiphiles with their corresponding molecular shapes in gray (described by the packing parameter P. The surfaces formed by their assembly will have varying degrees of curvature, and classical terms like mean (H) and Gaussian (K) curvature become useful in their characterization. **b.** A monolayer patch of DOPC with the pivotal point marked (just below polar region). The pivotal surface is roughly in line with the maximum lateral pressure brought about by interfacial tension (attractive) marked with γ. Lateral stresses derived from headgroup interactions and the steric repulsion of aliphatic chains are marked with C and S respectively. **c.** Lipid composition determines the overall electrostatic and mechanical properties of mixed lipid bilayers. Bilayer properties which can be tailored to study membrane protein function, structure, topology and insertion. Introducing lamellar (bilayer forming) and nonlamellar forming lipids alters the lateral pressure profile of a bilayer. Nonlamellar lipids with negative monolayer curvature (Type 2) such as PE, CL and ceramide, when added to planar bilayer forming lipid such as PC, PS, PG or SM form bilayers with decreased headgroup lateral pressure and increased acyl chain pressure. Conversely, when nonlamellar lipids with positive monolayer curvature (Type 1) such as PI, Lysophospholipids (e.g., LPC and LPA) and DAG are added to planar bilayer forming lipids, the result is a bilayer with increased headgroup lateral pressure and decreased acyl chain pressure. Lipid composition determines the overall electrostatic and mechanical properties of mixed lipid bilayers. Introducing nonbilayer forming lipids alters the lateral pressure profile of a bilayer. Electrostatic properties (via headgroup chemistry) on a bilayer can be modified by the addition of lipids with negatively charged headgroups (e.g., PG and PS) to their neutral counterparts (e.g., PC), altering the global net charge of the bilayer surface. **d.** Common membrane mimetics used in biochemistry. Depicted are detergent micelles, bicelles, liposomes and polymer–lipid nanodiscs.

In bilayer-based assemblies such as liposomes and bicellar constructs (detergent-stabilized bicelles, polymer nanodiscs, and membrane scaffold protein (MSP) nanodiscs), the complexities of membrane composition can be partly considered by employing different lipid mixes. Incremental changes in lipid composition allow us to derive the preferences and/or requirements of a given membrane protein for a particular membrane environment, influencing the downstream data interpretation. Amphiphiles, such as detergents and lipids, can be thought of as occupying distinct molecular shapes characterized by the packing parameter (P), a ratio relating the volume and hydrophobic chain length to the hydrophilic headgroup area. These shapes, arising from electrostatic interactions and rotational movements when aligned side by side, create monolayers with flat, positive, or negatively curved surfaces. Values of P classify amphiphiles as type 0, 1, or 2, respectively (Figure 5a).^97–99^ Molecular interactions of assembled lipids allow us to properly examine the tendency for an assembled monolayer toward a given curvature.

When two curved monolayers stack to create a bilayer with aliphatic chains pointing toward a midplane, they flatten and thin to eliminate energetically burdensome “void space”.^90^ Measured lamellar bilayer thicknesses are indeed thinner than the sum of two individual monolayers.^99,100^ This deviation from each monolayer’s preferred curvature creates a torque tension, which is the origin of stored elastic free energy (*g*_c_) in the membrane.^90,97–100^ Values of *g*_c_ vary with membrane composition, and the resulting lateral pressure profiles are experienced by any embedded membrane protein (Figure 5b).

This is most clearly demonstrated in minimal systems using membrane scaffolds like liposomes and bicelles of known composition. For example, membranes with increased lateral chain pressure (from nonlamellar lipid incorporation) reduce α-helical insertion during cotranslational folding.^101,102^ However, these “bulk” membrane properties are largely interconnected especially when combined with charge, pH, and hydration of individual amphiphiles. Therefore, careful selection of membrane mimetics and appreciation for the interdependency of physical properties is essential before drawing meaningful conclusions from analytical studies.^90,103,104^

### 3.2 Membrane Mimics

#### 3.2.1 Detergent Micelles

For membrane protein solubilization, common detergents include nonionic surfactants like n-dodecyl-ß-D-maltoside (DDM), NP-40 derivatives, and Tweens, as well as zwitterionic CHAPS and CHAPSO.^105,106^ These are considered less “harsh” than charged detergents like sodium dodecyl sulfate (SDS) because they avoid protein denaturation. However, the discrepancy between native lamellar membranes and normal micelles limits their use in kinetic studies of cotranslational folding. Indeed, membrane protein function is often compromised in micelles and only regained upon bilayer reconstitution. Despite this, the small size of detergent micelles makes them amenable to Cryo-TEM and NMR for structural elucidation.^90,91,93,94,106,107^ Structural biology is making concerted efforts to move toward scaffolds that better mimic native membranes, though eliminating detergents from membrane protein purification remains difficult.

#### 3.2.2 Bicelles

Bicelles are small (20–50 nm) planar discs (Figure 5d) of lamellar lipid bilayers composed of at least two amphiphiles: a lamellar-forming lipid and a detergent-like amphiphile such as CHAPS or DHPC. Detergents shield the hydrophobic rim from the aqueous environment; their positive mean curvature is suited to this role. The lamellar bilayer constitutes most of the bicelle (depending on the lipid-to-detergent ratio).^108,109^ Various bilayer and nonbilayer lipid compositions have successfully generated bicelles for iterative post-translational studies.^107–111^ Internal lipid dynamics (lateral diffusion) resemble liposomes, though membrane undulations are absent due to size constraints.^107^ While short-chain detergents concentrate at bicelle rims, some mixing with the lamellar portion occurs.^107^ Bicelle size, shape, and detergent composition directly influence membrane protein function, suggesting that biomimetic properties are dampened in smaller bicelles.^112^

Like micelles, bicelles are primarily used for post-translational characterization where membrane proteins are solubilized in micelles and reconstituted into bicelles, or detergent is added to proteoliposomes.^107–111,113–115^ However, bicelles with “softer” detergents (sterol cores with hydrophilic carbohydrate/phosphocholine moieties) have supported cell-free expression (Section 4.1) of bacteriorhodopsin, suggesting potential for cotranslational folding studies.^116^ Some promising applications of bicelles in mechanical unfolding studies have been reported, where a protein of interest, embedded within a bicelle, is tethered between two supports and force applied.^117^

#### 3.2.3 Liposomes

Liposomes (lipid vesicles) (Figure 5d) are frequently employed to study membrane proteins in biomimetic environments. These spherical vesicles consist of a single lipid bilayer enclosing an aqueous core, with diameters ranging from tens of nanometers to 100 μm.^118^ Liposomes enable reconstruction of defined membrane environments using synthetic lipids, allowing systematic exploration of major lipid classes: bilayer-forming lipids like phosphatidylcholine (PC), phosphatidylserine (PS), phosphatidylglycerol (PG), and sphingomyelin (SM) and nonbilayer lipids like phosphatidylethanolamine (PE) and cardiolipin (CL).

Liposomes facilitate study of membrane protein folding, structure, activity, and stability while enabling examination of specific lipid requirements and broader bilayer physical properties. This includes curvature, elastic stress, and lateral pressure profiles (Figure 5bc), which are effects known to influence insertion, folding, function, topology, and stability of integral α-helical membrane proteins.^119–124^ Nonbilayer lipids like PE are particularly instructive: they confer spontaneous curvature to each monolayer and, when frustrated within a planar bilayer, accumulate curvature elastic stress. This couples to a characteristic shift in the lateral pressure profile as increased pressure in the hydrophobic core from enhanced chain collisions and decreased pressure in the interfacial headgroup region.

These parameters can be precisely modulated through the compositional changes. Adding 1,2-dioleoyl-phosphatidylethanolamine (DOPE) tunes curvature toward the aqueous phase, while saturated chains (such as 1,2-dimyristoyl-phosphatidylcholine; DMPC) or single-chain lipids (such as lysophosphatidylcholine; lysoPC) suppress this tendency to curve. Incorporating saturated or lysolipids into unsaturated bilayers reduces the curvature stress and chain pressure while increasing the headgroup lateral pressure.^125^ This control makes lipid vesicles indispensable for elucidating the interplay between lipid composition, bilayer mechanics, and protein folding.

While informative for individual membrane proteins, these simplified mixtures lack the native membrane complexity.^126^ Even *E. coli* possesses thousands of distinct lipid species, with dynamic compositions varying across mammalian membrane regions: leaflets, organelles, and cell cycle stages.^127,128^ Despite technical challenges like light scattering artifacts,^129^ liposomes remain critical for membrane protein folding studies that would otherwise be impractical.

#### 3.2.4 Polymer–Lipid Nanodiscs

Preserving a native-like membrane is perhaps the best way to ensure correct membrane protein folding and activity. Copolymer-based nanodisc methodologies address this by directly extracting proteins from cell membranes within small, intact patches of their surrounding lipid bilayer (Figure 5d). In this approach, a planar nanodisc containing the protein of interest is excised from the membrane and stabilized by an amphipathic polymer, which forms a belt around the lipid patch through its hydrophobic side chains and hydrophilic backbone. The result is a stable polymer–lipid–protein complex that retains much of the protein’s native membrane context, enabling folding and stability studies under more physiologically relevant conditions.^130^ This preserves the structure and function of α-helical membrane proteins, yielding nanosized (10–20 nm), water-soluble, thermostable, monodispersed lipid nanodiscs.^131–135^ This methodology provides an important alternative to detergent micelles by offering a mimetic lipid environment that retains protein structure, function, and vital associated lipid interactions. It also eliminates free micelles in solution, which complicate biophysical and structural experiments, resulting in more stable protein–polymer complexes. In contrast, MSP methods require detergent solubilization before reconstitution into protein-belted nanodiscs, stripping associated lipids in a manner that may irreversibly impair function, as documented for the human serotonin transporter (SERT), where detergent treatment prior to liposome reconstitution caused transport loss compared to detergent-free polymer nanodisc methods.^136^ Lipid nanodisc methods have been employed to successfully purify several human membrane proteins, which nonexhaustively include the ABC transporters P-glycoprotein, MRP1, MRP4, ABCG2, CFTR,^137^ Adenosine A2a receptor (hA2aR),^138^ SERT^136^ and *de novo* artificial cytochrome CytbX.^139^

The use of polymer-based lipid nanodiscs to study membrane proteins began with styrene–maleic acid (SMA) copolymers, but the field has since expanded to include a broad and rapidly growing range of chemistries tailored to different experimental needs. For example, diisobutylene–maleic acid (DIBMA), in which styrene is replaced with diisobutylene, exhibits lower far-UV absorbance, reduced perturbation of lipid acyl-chain order, and greater resistance to cation-induced precipitation.^127,133,134^ DIBMA lipid particles (DIBMALPs) have also been reported to better preserve bilayer-like properties compared to SMA lipid particles (SMALPs).^136,140,141^ In addition, sulfonated and other modified derivatives have been developed to mitigate limitations associated with charged polymers, including interference with Ni-NTA binding and disruption of protein–protein or protein–lipid interactions.^142^

More broadly, the lipid nanodisc field has diversified substantially with an expanding toolkit of polymers that vary in charge, hydrophobicity, backbone chemistry, and stability. Recent comprehensive reviews describe the principles governing nanodisc assembly, lipid composition, size control, and their applications in structural biology, membrane protein biochemistry, and drug discovery.^130,135,143–146^

### 3.3 *In Vitro* Refolding of Membrane Proteins

Historically, membrane protein *in vitro* refolding studies have involved purified protein whose structure is perturbed with a chemical denaturant such as SDS or urea, subsequently removed using dilution, dialysis, or chromatographic methods, and unfolding and refolding followed using circular dichroism (CD) spectroscopy, differential scanning calorimetry (DSC), or cofactor binding to obtain kinetic and thermodynamic stabilities of folding within a membrane. A suitable membrane mimetic such as nondenaturing detergent micelles or liposomes is essential, adding a significant extra complication to the environment compared to that of soluble proteins.

Examples where these methods were employed include bacteriorhodopsin (bR),^147,148^ small multidrug transporter (EmrE),^149^ proton-galactose symporter from (GalP) and lactose permease from (LacY).^150,151^ *In vitro* folding studies were also performed using membrane proteins from cell-free platforms, including the potassium ion channel KcsA and Leucine transporter (LeuT).^152^ Typically, in these experiments the unfolded state, again in contrast to soluble proteins, must retain (or fold via) a significant α-helical secondary structure in order to fully recover the correct functional protein fold. Conceptually, this can be considered equivalent to the later stages of the three-stage model, where helix packing and other late folding events occur. These studies have allowed the measurement of thermodynamic stability of a variety of membrane protein structures. Mutagenesis and kinetic studies using stopped-flow techniques have been used to suggest important points of contact between helices and identify possible folding intermediates, as has been the case with bR,^153,154^ where helix packing parameters closely match the order of helix insertion and transition states observed *in vivo*,^155–157^ and are further discussed in section 4.3.1.1, marking perhaps the only work that directly links *in vitro* refolding to cellular TMD insertion and folding. Altering the lipid environment has allowed an examination of the effect of membrane parameters such as headgroup charge and lateral pressure profile on folding.^158^

Another method of studying the thermodynamic stability of membrane proteins has been steric trapping, where bulky streptavidin molecules are bound to the protein of interest to induce unfolding within the membrane. Refolding is promoted by outcompetition by fluorescent biotin labels.^159^ Application of the steric trapping method to bR was able to show its great kinetic stability in DMPC/CHAPS bicelles. The results suggested that its SDS-induced unfolded state would be unpopulated in the absence of SDS.^154^ Another extensively characterized membrane protein, rhomboid protease GlpG from *E. coli* has also been subject to this method. This time, GlpG was labeled with pyrene and placed in detergent micelles. The observations revealed a cooperativity network of unfolding, consistent with a previous magnetic tweezers (MT) study (see below).^159,160^ In another work, GlpG was placed into both DMPC:DMPG/CHAPS bicelles and liposomes of native *E. coli* lipids while being doubly bound to monomeric streptavidin and unfolded. Its refolding upon streptavidin removal was dependent on the lipid environment, with the *E. coli* lipids facilitating the interhelical interactions.^161^

As an alternative to chemical denaturants, single-molecule force spectroscopy (SMFS) has been used to dissect protein folding pathways under precisely controlled mechanical forces. Magnetic and optical tweezer (OT) experiments have enabled detailed characterization of membrane protein stability and folding landscapes by tethering their termini and monitoring force-induced unfolding and refolding transitions. For example, the TM domain of GlpG, which consists of six TM helices, has been the focus of several studies.^56,157^ Using MT to unfold GlpG reconstituted into DMPC/CHAPSO bicelles allowed the Bowie Group to build its folding landscape, where the protein inserts via rapid sequential helical hairpins with the rate-limiting step being the final two helices inserting. These experiments also show that removal of the lipid environment significantly reduced the force required to unfold it, further highlighting the relevance of the lipid environment for the stability of membrane proteins.^117,163^

Atomic force microscopy (AFM) has also been used to study membrane protein folding *in vitro* (reviewed extensively elsewhere^164^). AFM can be used to investigate refolding kinetics and thermodynamic stabilities and effects of lipid environment, ligand, and oligomerization of several proteins including bR,^165,166^ NhaA,^167^ and LacY,^168,169^ but it has so far not been used for cotranslational studies.

Hydrogen–deuterium exchange mass spectrometry (HDX-MS) is a technique that reports on local hydrogen bonding, and to a lesser degree solvent accessibility, as a function of time and therefore provides information on protein dynamics. It has been used in combination with polymer nanodiscs to measure the dynamics of membrane proteins in native lipid environments. A study on GlpG compared lipid conditions from two cells lines, BL21­(DE3), and C43­(DE3), which were grown at 16 or 37 °C while expressing protein to determine any observable changes in GlpG dynamics with lipid environment. Interestingly, where peptides corresponding to TM 2–6 were relatively well protected from HDX, part of TM1, and the linker region connecting the N-terminal, soluble, cytoplasmic domain, was unprotected and showed dynamic behavior. The lipid headgroup did not directly affect the HDX data; however, chain length and degree of tail saturation did.^170^

These *in vitro* studies and techniques provide an essential foundation as we move toward cotranslational experiments, where the entire pathway from membrane insertion to final folding can be examined in a more physiologically relevant context.

For a summary of recent examples of *in vitro* folding experiments and associated observations, see Table 1.

**Table 1:** Summary of In Vitro Membrane Protein Refolding and Stability Studies

Family	Protein	Method	Results	Refs
Microbial rhodopsin	Bacteriorhodopsin (bR)	- Time-resolved refolding kinetics using stopped-flow fluorescence and absorption	- pH dependent folding trajectory and role of retinal cofactor; rates of refolding and thermodynamic stability	^153,154,157,158,165,166,171^
		- Reversible folding measured by AFM	- Free-energy landscapes and folding kinetics. Helices unfold pairwise	
		- Phi-value and double mutant analysis.	- Helix B forms a major folding core, with helix G folding late, consistent with directional cotranslational folding (N- to C-terminus)	
Major Facilitator superfamily (MFS)	GalP	Reversible folding by fluorescence and CD	- Two state free energy of unfolding	^150^
			- Effects of lipid composition on folding and insertion	
	Lactose Permease (LacY)	- Reversible folding measured by fluorescence	- Demonstration of folding reversibility	^168,169,172–174^
		- Reversible folding measured by AFM	- Free-energy landscapes and kinetics. N-domain shows greater stability; domain flipping and αNPG ligand alters unfolding pathways. N-domain flipping was also observed in PE deficient membranes based on unfolding trajectory. YidC and SecYEG presence improve refolding efficiency	
	Glucose transporter 1 (GLUT1)	Thermal denaturation by DSC	Substrate binding increases thermal stability	^175^
Small Multidrug Resistance (SMR)	EmrE	Reversible folding by fluorescence and with lipids, ligand binding measured by isothermal titration calorimetry (ITC)	- Demonstration of folding reversibility	^149^
			- Effects of lipid composition on folding and insertion	
ATPases	CopA	Reversible folding by fluorescence and CD	Reversible system and free energy	^176^
	NaK-ATPase	Thermal denaturation by DSC	Thermal stability	^177^
	Ca-ATPase	Thermal denaturation by DSC	Thermal stability	^178^
ATP-binding cassette (ABC) transporters	Histidine permease (HisQMP_2_)	Reassembly of subunits	Noncooperative assembly of subunits	^179^
	Maltose transporter (MalFGK_2_)	Reassembly of subunits	Cooperative assembly of subunits	^180^
	Arginine transporter (Art(MP)_2_)	Reassembly of subunits	Assembly of subunits	^181^
	Vitamin B12 transporter (BtuCD)	Reassembly of subunits from folded and unfolded states	Cooperative assembly of subunits	^182^
MFS + ABC	DtpA, DgoT, MdfA, LacY; Ij1	Thermal denaturation by DSC	Stability/aggregation profiles; identification of stabilizing vs Destabilizing detergents	^183^
Na^+^/H^+^ antiporter	NhaA	Reversible folding measured by AFM	Demonstration of folding reversibility	^167^
Phosphoenolpyruvate:Sugar Phosphotransferase System (PTS)	Mannitol Permease (II^mtl^)	Thermal denaturation by DSC	Interactions of domains and contribution to stability	^184^
Anion exchanger (AE)	Anion Exchanger 1 (AE1)	Thermal denaturation by DSC	Interactions of domains and contribution to stability	^185^
Chloride-conducting ion channels (CIC)	CIC chloride transporter	Mechanical refolding by MT in bicelles	Unfolded into two independently folding halves	^186^
Rhomboid protease	GlpG	- Mechanical refolding using MT in bicelles	- Lipid composition is crucial to refolding efficiency; folding is from N- to C terminus direction, templated by α-helical hairpins	^159,162^
		- Steric trapping	- Cooperative network stabilizes native fold. The unfolded state is highly unstructured	
G-protein coupled receptor (GPCR)	β_2_-Adrenergic receptor	Mechanical refolding using magnetic tweezers in bicelles	Lipid composition is crucial to refolding efficiency; folding is from N- to C terminus direction, templated by α-helical hairpins	^162^

## 4 Toward Cotranslational Folding Studies of Membrane Proteins

### 4.1 Cell-Free Protein Synthesis

Cell-free protein synthesis, or *in vitro* transcription/translation (IVTT), allows proteins to be transcribed and translated entirely *in vitro*, making it an increasingly valuable tool for studying cotranslational folding and membrane protein insertion.^187^ IVTT is especially useful because it overcomes challenges often encountered in living cells, such as the high toxicity caused by overexpressing certain membrane proteins.^188^ Unlike cellular systems, IVTT does not require a cell wall or cell membranes, which allows researchers to directly manipulate and monitor protein synthesis in real time.^189^ This is perhaps the key advance in the field of cotranslational folding with synthesis directly coupled to translation to allow for extraction of the kinetic parameters driving the nonequilibrium process of cotranslational membrane protein folding (Figure 6). Monitoring membrane protein synthesis can be achieved by supplementing the reaction with a membrane mimic and incorporating radioactive labels^6^ or non-natural amino acids^190^ into the nascent proteins. Such systems enable real-time monitoring of nascent chain behavior (discussed further in Section 4.3.3.).

**Figure 6:**
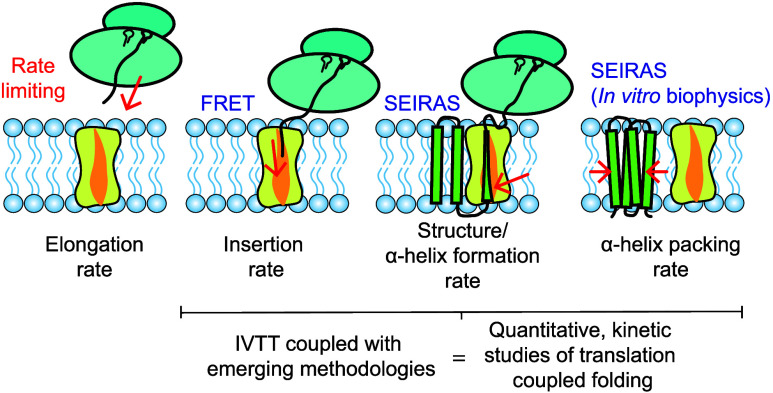
**IVTT for quantitative cotranslational studies.** Summary of rates associated with translation, structure formation, insertion, and folding in IVTT. When coupled with quantitative structural approaches, IVTT enables determination of the kinetic factors that drive successful protein folding within membranes. NB. the translocon has not been present in all published studies.

Table 2 highlights a summary of IVTT experiments used to understand favored lipid composition across different membrane proteins. This work has been reviewed extensively elsewhere.^91^

**Table 2:** Cell-Free Expression of Membrane Proteins Has Been Used to Identify Favored Lipid Compositions

Protein and organism		IVTT method and membrane	Results from study	Refs
BS-MraY *Bacillus subtilis*	10 TM dimeric enzyme for cell wall synthesis	S30 IVTT with nanodiscs	no preference for DMPC or DMPG	^213^
DsbB *E. coli*	4 TM disulfide bond reducing enzyme	PURExpress IVTT with liposomes	prefers low lateral chain pressure and neutral headgroups – DMPC	^6,214^
Bacteriorhodopsin (bR) *Halobacterium salinarum*	7 TM light driven proton pump	S30 IVTT with liposomes	prefers DOPC to DMPC, DPPC, DSPC – bilayer thickness and chain saturation important, DOPE inhibits	^215^
Connexin-43 *Rattus norvegicus*	4 TM hexameric small molecule channel	PURE system IVTT and liposomes	prefers DOPC, insertion decreases when DPPC or DOPG added	^216^
EC-MraY *E. coli*	10 TM dimeric enzyme for cell wall synthesis	S30 IVTT with nanodiscs	prefers DMPG to DMPC, needs 50% PG to function and form dimers	^213^
β1-AR *Meleagris gallopavo* (turkey)	7 TM GPCR	S30 IVTT with nanodiscs	prefers high lateral chain pressure and charge – PS or PG with unsaturated *trans* chains	^217^
endothelin B *Homo sapiens*	7 TM GPCR	S30 IVTT with nanodiscs	prefers high lateral chain pressure and charge – PS or PG with unsaturated *trans* chains	^218^
GlpG *E. coli*	6 TM rhomboid protease	PURExpress IVTT with liposomes	prefers high lateral chain pressure and charge – DOPG and DOPE	^6^
Opi3 *Saccharomyces cerevisiae*	4 TM (predicted) phospholipid methyltransferase	S30 IVTT with nanodiscs	preferred DOPG/DMPG to DMPG alone, DMPC not favored	^219^
MscL *E. coli*	2 TM pentameric mechanosensitive channel	S30 IVTT with liposomes	prefers high lateral chain pressure and charge – DOPG and DOPE	^220^
LacY *E. coli*	12 TM MFS secondary transporter	PURExpress IVTT with liposomes	prefers high lateral chain pressure and charge – DOPG and DOPE	^214^
XylE *E. coli*	12 TM MFS secondary transporter	PURExpress IVTT with liposomes	DOPG highly favored, DOPE also increases insertion yield	^214^
LeuT *Aquifex aeolicus*	12 TM (knotted) Neurotransmitter sodium symporter (NSS)	PURExpress and EXPRESSway IVTT with liposomes	poor insertion and does not vary with different lipid mixes. SecYEG does not increase insertion yield.	^188^
PCFT *Homo sapiens*	12 TM Proton-coupled folate transporter	*E. coli* S30 extract-based IVTT (Promega S30 T7 High-Yield) nanodiscs made with DMPC vs DMPG vs POPC	POPC nanodiscs did not yield soluble PCFT; both DMPC and DMPG supported soluble expression, with “highest level of soluble PCFT expression” in DMPC nanodiscs	^221^
pMMO (particulate methane monooxygenase complex; PmoA/PmoB/PmoC) methanotrophs	5 TM + 2 TM + 5 TM Metalloenzyme for biological oxidation of methane	*E. coli* lysate-based IVTT POPC nanodiscs compared to DMPC bicelles	POPC nanodiscs enabled soluble expression of all three pMMO subunits and gave best total + soluble expression in their screen; DMPC bicelles decreased total expression (∼25%) and gave minimal soluble expression	^222^
Zorya system proteins (ZorA, ZorB, ZorE; bacterial defense system components)	3 TM + 1 TM + 0 TM (per subunit)	*E. coli* TXTL (myTXTL) DOPE (supported lipid bilayers) SLBs vs DOPC SLBs; DOPE+cardiolipin SLBs; ECL SLBs (*E. coli* lipid extract)	ZorA/B/E showed preference for native membranes: no interaction with DOPC SLBs; stronger interaction with DOPE than DOPC; cardiolipin increased binding	^223^
Nipah virus membrane proteins (NiV F ΔSP and NiV G; single-pass TM proteins) Nipah virus	1 TM + 1 TM Viral membrane proteins	PURExpress with liposomes PC = 100% DOPC; PC/PE = 4:1 DOPC:DOPE; PC/PE/PS = 15:4:1 DOPC:DOPE:DOPS; MPLA titration in PC/PE/PS	For NiV F ΔSP, PC/PE/PS gave the largest increase in membrane association, PC/PE also strongly improved. For NiV G, PC/PE slightly outperformed PC/PE/PS. MPLA did not significantly change NiV F ΔSP or NiV G association	^224^
NS4B Hepatitis C	4 TM Viral membrane protein	Wheat Germ IVTT with detergent solubilization, reconstituted into liposomes	PC and PC/Chol gave best NMR spectra; DMPC was reasonable; DPPC yielded poor spectra	^225^
CrdS *Agrobacterium sp*.	7 TM Cell wall polysaccharide biosynthesis	Wheat Germ IVTT with cotranslational insertion into liposomes and reconstitution into nanodiscs	DMPC/DOPC/POPC and *E. coli* lipid extract supported synthesis; pure DOPG, DOTAP did not permit synthesis	^226^
Proteorhodopsin Marine proteobacteria ETB, IP, FFAR2, GPRC5B *Homo sapiens*	7 TM Light activated Proton pump, G protein-coupled receptors	*E. coli* S30 IVTT with nanodiscs (MSP1E3D1, DEPG, POPG, DMPC, DOPG)	Nanodiscs are more efficient than Salipro for cell transfer; transfer independent of nanoparticle lipid composition	^227^
bR, EmrE + 30 other MPs Various	7 TM (bR) + 4 TM (EmrE) Light-driven proton pump (bR), multidrug transporter (EmrE)	*E. coli* IVTT with DMPC nanolipoprotein particles vs DMPC liposomes	DMPC NLPs improve solubility; DMPC liposome efficacy different for different membrane protein	^228^
EsMscL-GFP *E. coli*	2TM (pentameric) Mechanosensitive ion channel	PURE IVTT with DOPC liposomes and polymer/lipid vesicles	Polymer/lipid vesicles improved MscL vs Pure DOPC; multipass rhodopsin showed different lipid dependence than MscL	^229^

Quantitative insight enables measurement of rates of membrane insertion, helix folding, and packing within the lipid bilayer, as well as translation elongation rates which are assumed to be the limiting factor for protein folding, suggesting some degree of post-translational rearrangement in the membrane. Kinetic parameters are only beginning to be determined experimentally, using fluorescence/FRET-based approaches for membrane insertion in the absence or presence of the translocon (Section 4.3.2) or SEIRAS to quantitatively observe helix formation as well as helix packing in the bilayer, including the effect of cofactors on folding as in the case with bR (see Section 4.3.3).

#### 4.1.1 Cell-Free Expression Platforms

Historically, most cell-free systems have been developed using crude cellular extracts, typically from *E. coli*,^191^ wheat germ,^192^ or insect cells.^193^ These extracts contain essential transcriptional and translational machinery, including ribosomes, tRNAs, aminoacyl-tRNA synthetases, and chaperones, supplemented with small-molecule cofactors such as amino acids and nucleoside triphosphates. The T7 RNA polymerase is a common addition to allow for expression from T7 promoters. For membrane protein synthesis, the reaction mixture must also include a biomimetic membrane component (Section 3.2). The stripped-back bottom-up approach reduces complexity, permitting observation using biochemical or sophisticated structural techniques. A detailed overview of cell-free expression systems across organisms can be found in ref 187.

One of the earliest extract-based systems, the *E. coli* S30 extract, provided the foundation for subsequent cell-free developments^191^ and has been used successfully for the production of both prokaryotic and some eukaryotic membrane proteins. One study successfully screened 61 different eukaryotic membrane protein targets using *E. coli* S30 extract against a matrix of six lipid mimetics in a 96-well format, identifying suitable production conditions for 35 targets (57%) in just a few days.^194^ There has been some notable recent success in utilizing machine learning to predict favorable lipid compositions for IVTT.^195^

Eukaryotic IVTT systems have also been developed, offering a unique combination of flexibility, control, and a native-like folding environment for complex, multidomain, and post-translationally modified proteins and membrane proteins.^196^ Higher eukaryote systems also provide chaperone proteins and native modification enzymes that are often imperative to correct protein folding and can be depleted prior to reaction using immunoprecipitation to measure the effect on protein folding.

The two predominant sources for eukaryotic cell-free extracts are wheat germ (*Triticumaestivum*) and insect cells, commonly derived from the fall armyworm *Spodoptera frugiperda* (Sf21). These systems have been used successfully to produce a range of important classes of human receptors, transporters, and channels.^194,197^ Each system offers distinct advantages; the wheat germ extract (WGE) is renowned for its high yields, robustness, and remarkable tolerance to a wide range of reaction conditions, including various salts and additives. This makes it exceptionally suitable for high-throughput screening, allowing for numerous expression conditions to be tested in parallel. Furthermore, WGE possesses the necessary chaperones and enzymes to facilitate the proper folding of complex eukaryotic proteins including the formation of essential disulfide bonds and post-translational modifications. Extracts prepared from Sf21 insect cells also contained endogenous ER-derived microsomal vesicles. These microsomes are sealed lipid bilayers that remain functionally active within the lysate. During protein synthesis, a nascent membrane protein can be cotranslated into the lumen or integrated directly into the lipid bilayer of these microsomes, effectively mimicking the natural biogenesis pathway within the ER.^196^ This provides an unparalleled environment for the correct folding, integration, and post-translational modification of the intricate membrane protein targets.

More recently, sophisticated mammalian expression systems have been developed for membrane protein expression, including rabbit reticulocyte lysate (RRL), HeLa, and Chinese Hamster Ovary (CHO) lysates. To boost protein yield in mammalian lysates, accessory proteins such as GADD34 and K3L, which relieve translational repression caused by eIF2α phosphorylation,^198^ can be supplemented. These systems can also employ vectors that utilize internal ribosome entry sites (IRES) to enable cap-independent translation initiation and improve translation efficiency.

Commercial eukaryotic lysates are now available, including the 1-Step HeLa IVT system (Thermo Fisher), RRL (Promega), and the Almost Living Cell Free Expression (ALiCE) system derived from *Nicotiana tabacum* BY 2 cell culture (LenioBio), which has been successfully used to produce a variety of proteins, including functional human CB2 rhodopsin-like GPCRs.^199^

As an alternative to extract based IVTT systems, purified systems such as the Protein synthesis Using Recombinant Elements (PURE) platform have since been developed and optimized for ‘clean’ expression of proteins, lacking cytoplasmic factors like molecular chaperones and modification enzymes. The PURE system, pioneered by the Ueda group,^3,5^ consists of entirely purified transcription and translation components; ribosomes, T7 RNA polymerase, aminoacyl-tRNAs, and translation factors, all derived from *E. coli*. This framework underlies several commercially available kits, including PURExpress and PUREfrex kits.^200,201^ The PURExpress kit features His-tagged purified components, whereas PUREfrex uses non-His-tagged components, enabling clearer detection and purification of target proteins.

#### 4.1.2 Kinetic Control of Cotranslational Folding

Different IVTT platforms offer complementary advantages. PURE systems are ideal for minimal, mechanistic studies requiring precise control over translation and folding and lipid composition and therefore over the membrane environment. Bacterial extract systems such as S30 provide higher throughput and yields, supporting more complex proteins while retaining some endogenous chaperone activity. WGEs are particularly suited for eukaryotic, multidomain proteins and can be scaled up for larger preparations. Finally, mammalian lysates, including HeLa, CHO, and RRL systems, excel at producing human proteins with native folding, posttranslational modifications, and biologically relevant activity. However, nascent chain elongation rates vary substantially between organisms and *in vitro* recombinant and extract-based IVTT systems. These differences have important consequences for the membrane protein insertion and folding.

In *E. coli*, elongation typically occurs at around 10–20 amino acids per second under optimal growth conditions of 37 °C,^202^ with eukaryotes generally translating approximately 10-fold slower at 3–8 aa s^–1^,^203^ though this can be largely tissue specific in higher eukaryotes.^204^ Many IVTT systems operate at even lower rates. For example, MRE600 *E. coli* ribosomes have been reported to elongate at 0.5 aa s^–1^ for a 233-residue soluble construct,^205^ approximately an order of magnitude slower than *in vivo* synthesis, but similar to rates reported in *in vitro* studies. Similarly, S30 extract systems show elongation rates similar to those observed in eukaryotic *in vitro* systems, yet still below typical bacterial *in vivo* rates.^206^

In fully reconstituted PURE systems, elongation can be slower still. Harris et al. report rates of approximately 0.13 aa s^–1^ at 30 °C based on SEIRAS measurements for GlpG and DsbB,^6^ These reduced rates are consistent with the dilute biochemical environment of cell-free reactions relative to the crowded cytoplasm, which limits macromolecular encounter frequencies and overall protein synthesis efficiency.^208^ Lowering temperature to 30 or 23 °C to further slow elongation does not necessarily alter folding outcomes in extract systems, indicating that elongation may not always be the sole determinant of folding efficiency;^203^ however, extract systems contain additional chaperone factors which may compensate for reduced translation efficiency.^209^ The Rodnina lab established an *in vitro* translation system reconstituted from purified *E. coli* components that performs at near *in vivo* rate and accuracy,^210,211^ allowing them to monitor cotranslational membrane insertion of substrates such as LepB and EmrD within biologically relevant time frames.^11^

For membrane proteins, elongation rate shapes the timing of helix emergence, SRP recognition and translocon mediated insertion. When elongation is substantially slower than it is *in vivo* (10–20 aa s^–1^ in *E. coli)*, as observed using PURExpress (0.4 aa s^–1^), this can have several consequences on folding, as membrane protein folding is intrinsically cotranslational and kinetically partitioned. Therefore, deviations from physiological elongation rates and from coordinated chaperone activity can significantly reshape folding trajectories and may potentially expose the nascent chains for longer prior to membrane engagement, leading to non-native interactions.

Codon usage may also modulate local translation elongation rates *in vivo*, where translation is not uniform along the mRNA sequence, with events of rapid translation, followed by pausing.^207,212^ In reconstituted systems, tRNA pools are simplified, and this level of translational tuning may not always be a factor.

Ultimately, careful consideration of elongation kinetics is therefore essential when interpreting membrane protein folding outcomes in vitro, further exemplifying the need for quantitative studies of folding within membranes.

### 4.2 Ribosome-Bound Nascent Chain Complexes

A major advance to understand structured intermediates formed during cotranslational folding has been the development of artificially stalled ribosome–nascent chain complexes (RNCs) (Figure 7a). RNCs enable proteins to be stalled at defined translation points without dissociation from the ribosome. Stalling is typically introduced between structural elements (e.g., domains or TMDs), generating a series of progressively longer constructs up to the full-length protein.^13–21^ Purified RNCs can then be analyzed by advanced structural and biophysical tools to provide high-resolution insight into the structure and dynamics of emerging polypeptides.^14,16,230,231^ By capturing discrete “snapshots” of nascent chains as they exit the ribosome, RNCs may reveal folding intermediates or transient states which are formed during synthesis, bridging the gap between *in vitro* structural studies and transient cellular events. This strategy is particularly powerful for membrane proteins, where it enables investigation of insertion and folding within a native-like lipid environment.^13^

**Figure 7:**
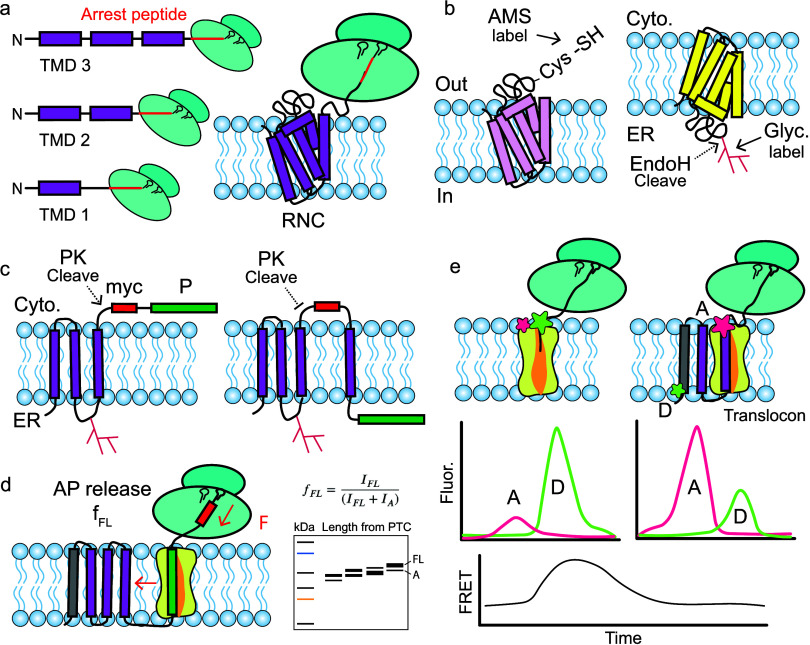
**Biochemical toolbox for membrane protein cotranslational study.** **a.** RNC generation through specific placement of an arrest peptide sequence. **b.** Topology measures of proteins at the membrane utilizing cysteine alkylation with AMS, or presence and/or cleavage of ER/Golgi decorated sugars on membrane proteins by EndoH. Mass shifts for both AMS labeling and sugar cleavage are usually observed by SDS-PAGE. **c.** Proteinase K based limited proteolysis also measures topology of nascent chains via introduction of the myc and P tags. Cleavage of these tags occurs if they’re facing the cytosolic side. **d.** Schematic of FPA. An arrest peptide (red) can be released if the nascent chain generates a large-enough force induced by a folding event or translocon integration to release ribosome stalling. This is quantified using f_FL,_ which is the ratio between the full-length (FL) and arrested band (A) on an SDS-PAGE gel. **e.** FRET pairs (A: acceptor; D: donor) can be included on the emerging nascent chain and the translocon. FRET between the nascent chain and SecYEG can indicate kinetics of insertion^11^ but, currently, cannot be used to observe folding within the membrane.

RNCs are often generated in the cell based on natural arrest peptide sequences; this has been performed in both bacterial and mammalian cells.^13,14,232–234^ Arrest peptides are short stretches of polypeptides that stall translation, usually by establishing stable contacts within the ribosomal tunnel wall, which distorts the shape of the ribosome peptidyl transferase center (PTC), preventing the formation of subsequent peptide bonds. To generate bacterial RNCs, SecM-based stalling sequences are most commonly used, yet other options such as the tryptophan-dependent stalling sequence of TnaC^234–237^ have been used to generate membrane protein RNCs. To generate human soluble RNCs, researchers have utilized hCMV uORF2 (which utilizes eRF1 rather than PTC perturbation),^238^ or wild-type^232^ or enhanced^14^ Xbp1 stalling sequences, but to date no mammalian stalling sequences have been used for membrane proteins. Other methods have been used to generate soluble RNCs for biochemical studies using cell-free expression *in vitro*,^19,238–244^ including inhibitory codon pairs, amino acid depletion, poly­(A) tracts and truncated mRNAs, which have all been utilized to isolate RNC’s cell-free systems.^231,245–247^

Unsurprisingly, more RNCs have been reported for soluble than membrane proteins for nascent chain interrogation; both present challenges, including preserving ribosome integrity and retaining nascent chain attachment.^19^ Membrane protein RNCs add further complexity due to the need for a membrane environment that may contain partially inserted TMDs.^40^ A key consideration is the solubilization strategy, typically involving detergent,^40^ reconstituted nanodiscs,^248^ or polymer nanodiscs.^249^

Purified RNCs have primarily been used in conjunction with cryogenic electron microscopy (Cryo-EM) to capture structures of ribosome-translocon complexes for mechanistic studies of the cotranslational membrane insertion of membrane proteins, though this has proven to be incredibly challenging due to the inherent conformational heterogeneity with these large transient complexes. A range of *E. coli* RNCs of GlpG, FtsQ, proteorhodopsin, CyoA and OmpA have been purified in detergent micelles.^13,235^ The 7 TM Proteorhodopsin (PR) was used to generate SecY-bound RNCs in *E. coli* with the tryptophan dependent TnaC stalling peptide.^235^ This resulted in a 2 TM helix RNC with a cytoplasmic loop, designed for tight interactions with SecY. RNCs and SecY were copurified in detergent, and an RNC-SecY structure was resolved with Cryo-EM to 7.3 Å. Interactions between the positively charged cytoplasmic loop of the first two helices with a ribosomal rRNA helix were also observed, suggesting a role for the ribosome in retaining charged loops on the cytoplasmic side of the bilayer during TM integration into the membranes in this system.

A major limitation of these studies is the stripping away of native lipids during the detergent solubilization and purification process, which are important for promoting the correct function, stability, and cotranslational folding of membrane proteins.^40,85^ MSP nanodiscs, which provide a customizable lipid environment, have also been used to generate RNCs.^40,85^ Using this approach, Cryo-EM structures of the FtsQ nascent chain inserted into SecYEG reconstituted in nanodiscs have been resolved, enabling visualization of the translocon conformation within a bilayer environment during the early stages of nascent-chain insertion. The nascent chain itself, however, was not resolved.^250^ Furthermore, in a recent study of FtsQ-LacY chimera, SecY was shown to promote local unfolding and refolding of helices in different transposon states by stabilization by a hydrophilic groove between SecY TM3 and 4, outside of the channel and within the lipid environment, suggesting an active SecY chaperoning ability for polytopic TM insertion. When these helices were disrupted, it led to global membrane protein disruption.^251^ Busch et al. have recently showed that NuoK nascent chains can drive the assembly of the YidC-SecYEG complex for correct folding in MSP-nandiscs^230^ and suggest an insertion model more similar to the eukaryotic Sec61-GEL-BOS mechanism for polytopic proteins.

Unfortunately, although MSP-nanodiscs themselves provide a lipid bilayer context for the RNC sample, the preparation of them still relies on detergents to originally extract the complex from native membranes before assembly into the nanodisc. Polymer nanodiscs, prepared using the DIBMA copolymer, were used to purify GlpG RNCs directly from the *E. coli* membrane, maintaining the native lipid environment^13^ throughout the purification process. While polymer nanodiscs are an exciting prospect for studying RNCs, they can be practically challenging. Polymers typically have lower membrane solubilization efficiencies than detergents, and not all are able to tolerate the divalent cations or high salt buffers necessary to ensure ribosome integrity,^252^ often leading to a necessity for polymer screening during sample optimization. However, many polymers have now been developed (Section 3.2.4) and are a promising tool to build the complexity of RNC samples toward that of the true *in vivo* folding environment.

### 4.3 Interrogation of the Emerging Nascent Chain

#### 4.3.1 Biochemical Studies

##### 4.3.1.1 Cysteine Accessibility, Glycosylation and Protein Topology

Early techniques aimed to track the TM insertion order over time by monitoring the translocation of a unique extracellular cysteine across the cytoplasmic membrane *in vivo*.^155^ The membrane impermeable 4-acetamido-4′-maleimidylstilbene-2,2′-disulfonic acid (AMS) attaches to translocated cysteines extracellularly, shifting protein motility in an SDS-PAGE^253,254^ (Figure 7b). Combined with ^35^S-methionine pulse-chase labeling, insertion rates of polytopic membrane proteins can be tracked; this was utilized to investigate the insertion pathway of bR and revealed that N-terminus translocation and first TM helix insertion occur cotranslationally, followed by translocation of the BC loop.^155,156^ However, it was shown that FG loop translocation occurred post-translationally, suggesting a sequential order of cotranslational TM insertion, and consistent with previous *in vitro* studies (Section 3.3).

In other work, cysteine labeling was combined with antibody epitope binding to show that lipid composition has the ability to influence TMD topology with altered *E. coli* lipids.^255^ LacY was expressed in *E. coli* cell strains where the proportion of the dominant lipid type, PE, could be manipulated. Cysteine labeling of the periplasmic and cytoplasmic loops showed that the absence of PE causes an inversion of the first 6 TM domain of the 12 TM LacY in the membrane, consistent with the loss of binding of an antibody that recognizes the native fold. The correct topology could be recovered post-translationally by increasing PE levels to normal amounts.^255,256^

Glycosylation can also be used to assess protein topology co-/post-translationally. Simply, monitoring protein glycosylation in the Golgi can be used as a proxy for correct folding and trafficking from the ER.^257^ Kim et al. (2005) mapped the topology of the eukaryotic polytopic membrane protein STT3, using C-terminal reporter fusions and insertion of glycosylation sites in the loops between predicted TMs.^258^ This was used to infer orientation; if the inserted site was glycosylated by the cell machinery, it resides in the ER lumen, whereas if it was not glycosylated, it was in the cytosol (Figure 7b). They subsequently predicted that STT3 had 11 TMs with an N_cys_-C_lumen_ orientation. A later study expanded on this, by analyzing the insertion efficiency of STT3 predicted TMDs using an *in vitro* asparagine-linked glycosylation assay.^259^ Using the reporter protein leader peptidase (Lep), which contains TM helices that can be replaced with any sequence of interest, they analyzed each STT3 TMD and monitored the efficiency of translocon-mediated membrane insertion by the glycan modification of the engineered receptor sites within Lep. Subsequently, they were able to elucidate that STT3 likely has 13 TMDs rather than 11. A recent study has built upon the aforementioned glycosylation assay to engineer glycosylation sites around test peptide sequences inserted into LepB to differentiate between 3 states: luminal (water-soluble), interfacial, and transmembrane based on the resultant glycosylation pattern.^260^

##### 4.3.1.2 Limited Proteolysis

Another popular biochemical method to interrogate the solvent accessibility of helices and nascent chains as a proxy for membrane insertion and protein topology is limited proteolysis^261^ (Figure 7c). This, however, cannot directly report on folding within the membrane. The technique is largely a post-translational measure of helix insertion, though there has been limited use to track the insertion of artificially generated nascent chains that can relax into their steady state, to generate a low-resolution picture of folding in the membrane. Usually, nascent chains are assayed by partial digestion of the RNC using enzymes such as proteinase K or trypsin, with unfolded populations being susceptible to digestion and the folded ones protected, or a reporter epitope being accessible or not, highlighting orientation in the cell. This approach has been used to study a range of soluble and membrane proteins, with early examples including dihydrofolate reductase (DHFR),^262^ firefly luciferase (FLuc),^263^ and *E. coli* OmpR.^264^

Aquaporin (AQP) water channels are a family of eukaryotic polytopic membrane proteins,^265^ and their topology throughout cotranslational folding has been investigated by limited proteolysis.^266,267^ Lu et al. (2000) used epitope tagged constructs of AQP1, which involved sequentially truncating the nascent chain and attaching it to a protease reporter domain (c-myc epitope tag or passive C-terminal translocation reporter (P)).^266^ The location of the reporter domain in the cytosol or the ER lumen indicates the orientation of the respective TM; therefore, by characterizing the protease accessibility of both reporters in nascent chains of increasing length, the topology of the resultant TMs can be defined throughout the various stages of translation. This work revealed that after synthesis of TMs 4–6, TM3 underwent a 180° rotation, leading to C-terminal residues flanking TM3 being translocated from the cytosol to the ER lumen, while N-terminal residues flanking TM3 are translocated in the opposite direction. This event pulls TM2 and 4 into the bilayer in a conformation consistent with 6 TM topology. Further investigations into the homologous AQP4, using limited proteolysis on chimeric AQP1/4 constructs, highlighted how minor sequence variations across homologous polytopic membrane proteins can significantly affect the folding pathway, as AQP4 has three internal signal anchor sequences and three stop transfer sequences that initiate and terminate TM translocation, respectively.^267^ AQP4 achieves its 6 TM topology in this vectoral and cotranslational manner, a clear difference to AQP1.

Another protein that has been extensively studied with limited proteolysis is the cystic fibrosis transmembrane conductance regulator (CFTR).^268^ Kleizen et al. (2005) combined limited proteolysis with *in vitro* translation to assess the protease susceptibility of elongating nascent chains in comparison to the fully folded protein.^257^ They found that all CFTR domains reached a protease-resistant state during nascent chain elongation, strongly indicating a cotranslational folding model. A more recent study by the Braakman group combined kinetic radiolabeling in live cells with antibody epitope mapping and protease susceptibility assays to reveal the sequence of events that make up the folding pathway of CFTR.^269^ They established two distinct stages of folding; the first occurs cotranslationally, where the two transmembrane domains (TMD, each containing 6 TM helices) and the first nucleotide-binding domain (NBD1) fold as individual domains to near-native structures. The second stage occurs post-translationally, whereby protease resistance increases for both NBD2 as it folds and the TMDs as they assemble together with NBD1 to form the final structure. A recent preprint from the Braakman group explores a new corrector to rescue the misfolding of the cystic fibrosis causing F508del-CFTR mutant.^270^ Using radioactive pulse-chase experiments and limited proteolysis, they tracked the folding and assembly of each CFTR domain and characterized the novel X307810 corrector. They observed that X307810 changed the protease resistance of the linker between TMD1 and NBD1 during the late stages of CFTR folding. This is the first time that a CFTR corrector has added a new fragment to the typical limited proteolysis pattern.

##### 4.3.1.3 Force-Profile Analysis

Another method to study helix insertion and the role of the translocon in cells is the use of arrest peptides for force profile analysis (FPA) (Figure 7e).^271^ When translated, these peptides stall polypeptide synthesis; utilizing their natural function of regulating nascent chain translation, they can act as an *in vivo* force sensor when introduced to other proteins. Interactions between the nascent chain and the translocon or partitioning of a TMD into the membrane can exert a strong enough force to allow translation to overcome stalling to release the nascent chain from the ribosome.^271,272^ When used to map a protein of interest at residue-level resolution, FPA is a proxy for an approximate free energy of membrane protein insertion.^273,274,9,233^ In principle, this method is best suited to measuring first-stage folding. However, since interactions between the neighboring parts of the expressed protein can also influence the pulling force, insight into second-stage folding can also be gained.^274^ Placement of the stalling peptide into different positions of the sequence of the target protein permits the construction of a force profile by measuring the fraction arrested protein (A) relative to full-length protein (FL), typically observed using SDS-PAGE.

Introduction of the SecM peptide into the sequence of Lep was initially used to show that a pulling force was generated by insertion into the membrane through the SecYEG translocon *in vivo*.^273^ In this same work, it was shown that introduction of charged residues, or a proline that disrupts α-helical formation, reduces the pulling force of insertion.^273^ Lep has also been used to demonstrate that charged residues can experience a pulling force arising from transmembrane electric potential, and from the interactions with the membrane surface.^272^ More recently, Lep was used to assess the sequence-dependent tendency of transfer from the translocon to the extramembrane space, an interfacial conformation, or into a TM segment.^260^

Polytopic membrane proteins, CaiT, NhaA, EmrD, BtcU, and GlpT, have all been used with FPA and show that N-terminal TM helices can aid the insertion of C-terminal helices as they exit the translocon.^274^ This effect was further demonstrated in a systematic scan of three proteins with varying numbers of TM helices: EmrE (4 TM), GlpG (6 TM), and BtuC (10 TM), to improve our understanding of the importance of charged residues, re-entrant loops, surface helices, and point mutations for correct protein folding.^9^ The consequences of these features, together with the additional observation of α-helical hairpins which drive insertion, was further highlighted using voltage-sensitive channel KvAP.^10^

Although FPA has revealed important details about the steps involved in polytopic membrane protein folding, it does not provide direct kinetic information since it depends on intermediates created by programmed ribosome stalling. Therefore, the precise timing and order of folding events cannot be ascertained.

#### 4.3.2 Fluorescence-Based Approaches

Fluorescence methods have been applied to study cotranslational RNC interactions with the translocon, and insertion of membrane proteins.^275,211,276–278^ FRET (Förster resonance energy transfer) has been used to monitor cotranslational insertion of membrane proteins through the Sec translocon (Figure 7e).^11^ In this approach, an acceptor fluorophore (BODIPY-labeled fMet) was placed on nascent chain N-termini, while donor fluorophores were positioned at either cytoplasmic or periplasmic loops of SecY via cysteine-maleimide chemistry. For type I membrane proteins (N-out topology), when the donor was placed cytoplasmically, the FRET signal showed an initial increase followed by a slower decrease, suggesting the acceptor “passed by” the donor position. When the donor was positioned periplasmically, the increase was delayed and no decrease occurred. Conversely, for type II membrane proteins (N-in topology), no decrease occurred in either case and kinetics were delayed. These results support the ‘headfirst’ insertion model for type I membrane proteins and highlight the ribosome’s role in holding the first transmembrane segment while the second emerges for type II proteins, mediated through interactions with charged nascent chain residues.

Single-molecule FRET has also monitored the dynamics of the SecYEG translocon lateral gate.^12^ With SecY labeled at opposite ends via cysteine-maleimide chemistry, conformational fluctuations were reflected in the FRET signal. Results revealed the gate’s highly dynamic nature and showed that transmembrane insertion increases sampling of the open state. Fluorescence-based approaches have tracked how nascent chains are recognized during translation. Changes in fluorescence of labeled SRP were followed to determine association affinities with ribosome-nascent chain complexes.^279^ Results showed that SRP affinity was much greater for loaded ribosomes than empty ones.

Single-molecule FRET monitored the association of NAC with SRP,^69^ using donor-labeled NAC and acceptor-labeled SRP via cysteine-maleimide chemistry. Results supported a model in which SRP is captured by NAC before stable association with the ribosome and nascent chain. FRET has also been used to study SRP association with ribosome-nascent chain complexes displaying signal-anchor sequences.^280^ Further FRET studies revealed SRP rearrangements in response to signal-anchor sequence emergence.^281^

While these studies have provided valuable insights into some cotranslational processes associated with the translocon, direct monitoring of nascent polypeptide folding within the membrane remains challenging. Current approaches rely on labeling the ribosome, translocon, or chaperone machinery rather than the nascent chain itself. While these strategies have enabled recent, elegant single-molecule studies of chaperonin interactions with soluble RNCs using total internal reflection fluorescence (TIRF) microscopy,^282^ the site-specific incorporation of single fluorophores into nascent chains, either through non-natural amino acid incorporation or post-translational chemical labeling, remains technically challenging. This is particularly true for membrane proteins, and consequently, direct observation of cotranslational folding dynamics within bilayers using FRET has not yet been possible.

#### 4.3.3 Surface-Enhanced Infrared Absorption Spectroscopy

Surface-enhanced infrared absorption spectroscopy (SEIRAS) monitors secondary structure formation of membrane proteins cotranslationally, in real time as they are being expressed using cell-free systems (Figure 8). SEIRAS captures some of the most kinetically rich information on membrane protein folding and yields a structural fingerprint of protein biogenesis, in contrast to the steady-state low-resolution biochemical and structural approaches described above. SEIRAS offers unprecedented access to transient and previously elusive conformational states, achieving a temporal resolution that surpasses those of other available methods.

**Figure 8:**
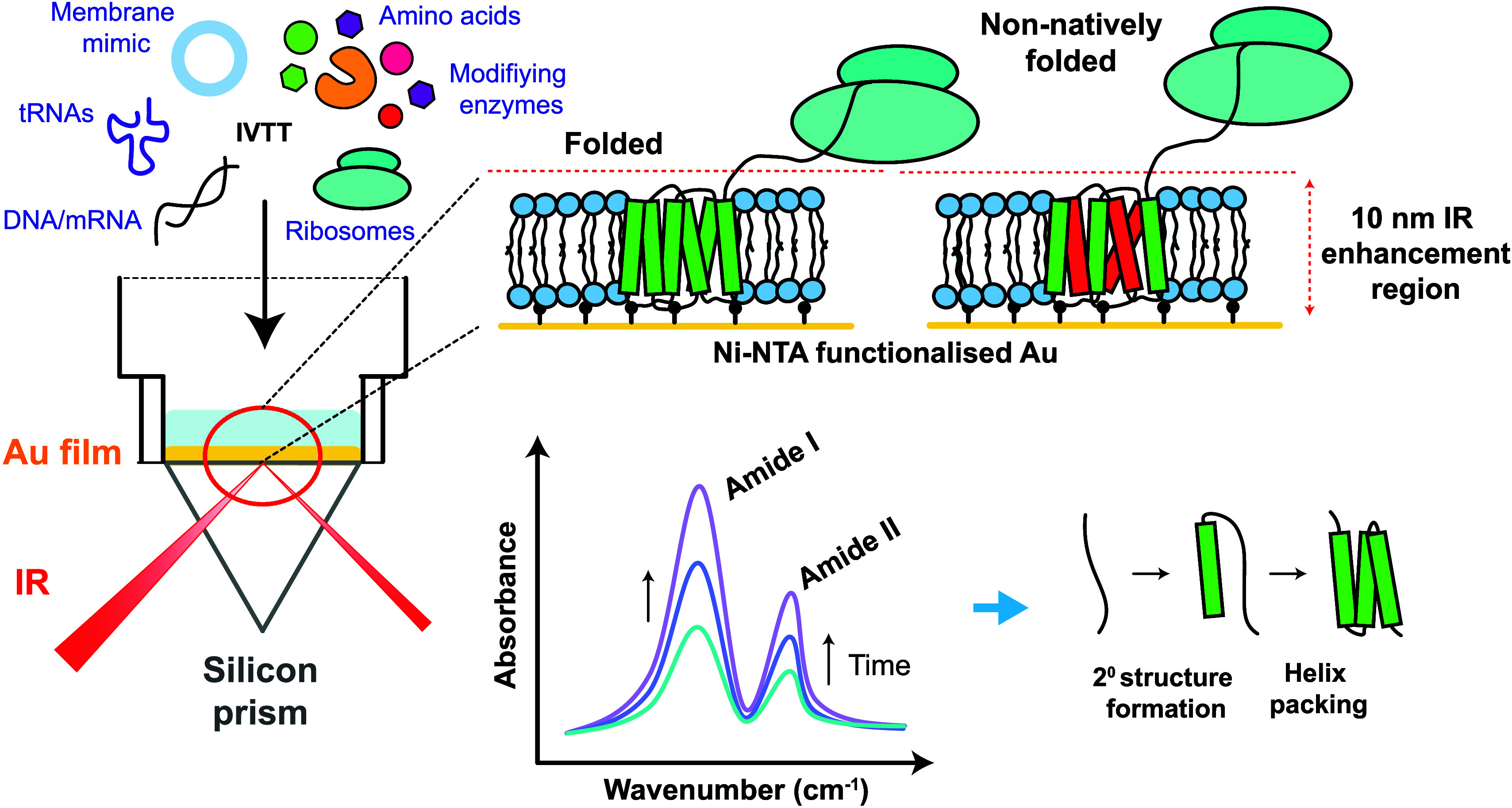
**SEIRAS and experimental setup.** Global setup of the SEIRAS reaction.^254^ IVTs synthesize MPs into membrane mimics of specific lipid mixes, which are deposited onto the gold coated stage. Synthesis happens within the 10 nm enhancement region, and signals outside of this, attributed to the cell-free lysates, are invisible to IR, allowing measurement of the nascent chain alone. Example data shows the increase of Amide I and II bands with time as protein is synthesized and acquires structure. Peaks can be integrated to deconvolute the secondary structure component, informing on the formation of secondary structure and helix packing.

A thin gold layer deposited on a silicon chip or prism enhances the IR signal of molecules within 6–10 nm of the surface by 10–100-fold compared to conventional IR.^283^ Only signals within this 10 nm are enhanced, so that when a membrane is deposited on the gold surface, water and IVTT components above that membrane are, effectively, nearly IR invisible. This is a major advantage of this work, as it enables the observation of signals arising from the membrane only during protein expression. Furthermore, fast IR spectrometer acquisition times enable a spectrum recording, depending on the instrument and detector, every 5–10 s. Again, depending on the IVTT system used and the temperature, this equates to approximately every 1–10 amino acids translated, so that this technique can allow the real-time monitoring of protein folding.^7,8,6^ Spectra can typically be deconvoluted from around 3 min after initiation of protein synthesis and continue for several hours to track much later folding events. Analysis primarily of the amide I band (mainly C=O stretch, 1600–1700 cm^–1^)^284^ retrieves information on a relative abundance of secondary structural components, including accumulation of helical structure, and changes to the position and width of this band can indicate helix formation packing during cotranslational insertion.^284^ The amide II band (C–N stretching and N–H bending) intensity is typically smaller due to the orientation of a-helices inserted into the bilayer, but it can be used to report on changes to peptide orientation or accumulation of nonhelical structures. Together, these give each protein a structural fingerprint. The signal-enhancing gold surface can also tether nanodisc monolayers via S–Au bonding.

Baumann et al. (2016)^8^ first demonstrated this approach as a label-free method for observing membrane protein folding in real-time. Using SEIRAS, they monitored the cotranslational insertion and folding of bR into DMPC MSP nanodiscs immobilized on a gold surface. The authors revealed 4 distinct folding stages for spontaneous bacteriorhodopsin (bO) (bR without retinal cofactor) insertion and folding: 1) clearance of preadsorbed molecules (0–10 min), 2) membrane insertion of unstructured polypeptide (10–60 min), 3) An increase and saturation of amide band intensity over the next 4 h, indicative of secondary structure formation (α-helices), which 4) coincides with a narrowing of amide I band area, indicative of tertiary structure assembly (helix packing). Critically, retinal cofactor presence during insertion was essential for efficient and correct folding as previously shown,^154,285^ and without it, bR fails to adopt the native conformation.^8,286^

Subsequent SEIRAS studies have expanded our understanding of cotranslational folding mechanisms. GlpG and DsbB inserted and folded unassisted into tethered DMPC nanodiscs, demonstrating that membrane protein insertion can be driven solely by residue hydrophobicity, tuned by membrane lipid composition.^6^ SEIRAS revealed DsbB’s notably slower folding compared to other proteins, remaining more disordered, before adopting a predominantly α-helical fold after translation exhaustion; suggestive of a post-translational final folding event. This is likely a collapse of helices 2 and 3 (of 4) based on hydropathy predictions.^6,254^ In contrast, GlpG, like bR (without the requirement of retinal) folds cotranslationally, likely driven by its favorable thermodynamic properties and consistent with MT studies^162^ and molecular dynamics simulations^287^ (Section 3.3). Direct comparison of cell-free synthesized versus recombinantly produced GlpG showed highly similar overall secondary structures by FT-IR spectra, though subtle differences appeared in β-turn structure bands, which were shifted toward higher wavenumbers in cell-free protein (<1640 cm^–1^ versus >1670 cm^–1^), indicative of the presence of misfolded and/or aggregated protein.

More recent work studied bR and three other bacterial rhodopsins.^7^ While initial α-helical fractions during membrane insertion were similar across all four proteins, only bR displayed spectral features characteristic of compact α-helical tertiary structure formation. The other rhodopsins failed to fold, mirroring bR’s failure without retinal, probably due to reduced TMD hydrophobicity hampering retinal association.^7^

These studies demonstrate that the proteins examined insert into membranes through hydrophobicity-driven spontaneous insertion. However, the spectra consistently indicate the presence of some misfolded structure, which may be reduced if translocons were incorporated into the membrane system. Identification of which regions of the polypeptide contribute to these misfolded signals would be informative; however, SEIRAS does not provide residue-level resolution and therefore cannot directly determine the identity or order of helix formation without additional labeling strategies. A further constraint on protein insertion could be access to the bilayer surface, limited to the area available on the prism-interacting face. Despite these limitations, the time-resolved nature of SEIRAS is a major advantage. Changes in the amide I and II bands allow the kinetics of secondary structure formation and helix packing to be followed in real time during membrane insertion, making it one of the few approaches capable of monitoring both structural acquisition and folding rates as the process occurs.

## 5 Toward Computational Nascent Chain Modeling

Computational methods for predicting protein structures have advanced significantly, culminating in the 2024 Nobel Prize in Chemistry for teams behind AlphaFold^26^ and RosettaFold.^288^ Deep learning methods, spearheaded by AlphaFold, dominated the 14th Critical Assessment of Structure Prediction (CASP14) event,^27^ with impressive early successes in protein structure prediction and design.^289–292^ However, experimental techniques like X-ray crystallography, NMR, and cryo-EM used to determine structures deposited in the Protein Data Bank^293^ often neglect or neutralize lipid effects due to experimental constraints. Deep learning methods trained on these structures similarly neglect lipid effects, meaning initial predictions for membrane protein structures can differ significantly from their dynamic cellular conformations.^294^ Since membrane protein structure determines function and depends on both environment and local free energy landscape motions,^295^ methods that use these predictions as starting points while capturing dynamic behavior to explore conformational space are increasingly important.

There are several simulation techniques that can be employed to this end; Monte Carlo simulations involve a rate of transition between predicted or known states and have been used to explore the cotranslational folding pathway between non-native intermediate structures with an implicit ribosome and examine cotranslational protein folding for smaller, soluble peptides.^296^ When a topology-based simulation model, rather than a dynamics-based one, is utilized, the faster folding speed means that folding pathways can be explored in more detail,^297^ including quantification of the misfolding propensity of different proteins.^298^ However, these methodologies are primarily statistical rather than physical and thus neglectful of the interatomic forces that govern the transition between macroscopic structures. A technique particularly suited to examining the interplay between membrane proteins and their environment is molecular dynamics (MD),^299^ which can look at the atomic-level flexibility of membrane proteins and how their dynamic tertiary structure can change under different conditions.^300,301^

MD simulations for protein folding were originally limited by the need for a) accurate models describing the atoms and how they interact, b) a sufficiently long simulation so that the conformational space is appropriately sampled, and c) robust and effective data analysis.^302^ The first of these issues was addressed with the development of more accurate force fields informed by quantum chemistry calculations and validated by experimental measurement,^303–306^ and the third was addressed by the development of software packages that were designed with the analysis of MD simulations in mind, which (nonexhaustively) include PyLipID,^307^ LiPyphilic,^308^ MDTraj,^309^ and MDAnalysis.^310^ Sampling the system for a sufficiently long time has been addressed by the decrease in cost and increase in power of computing hardware,^311^ as well as through initiatives like Folding@Home.^312–315^ The mpstruc database^316^ is a curated repository that collects peer-reviewed membrane protein structures for further utilization, ensuring that these structures are available for communal use under FAIR (findable, accessible, interoperable, reusable) principles. These factors together have ushered in a new regime in what is possible for MD and positioned it at the forefront of structural modeling of complex biological systems. The significant effect of local environment on membrane protein structure and function remains critical.^317^ MD is the technique best positioned to examine the interactions between proteins and lipids at an atomic level^318^ as well as explore folding pathways in a detailed way at the appropriate time scale.^319^ It has been employed on protein structures since the 1970s, and MD continues to explore systems with increasingly higher complexities and biological relevance.^320,321^

Explicit MD simulations of the Sec translocon translocating and integrating nascent proteins remain as yet beyond reach due to required time scales,^322^ though MD has elucidated SecYEG mechanisms^323^ and identified residues and motifs critical to function through comparative mutant studies.^324^ Direct simulation of full binding, translocation, and insertion processes will likely first be achieved using coarse-grained (CG) methods, which employ united-atom approaches to reduce calculations per time step,^325^ and smooth the free energy landscape, allowing longer time scales to be explored.^326^ This approach has already explored SecY-cardiolipin interactions.^327^ While explicit ribosome representation proves challenging due to size,^328^ notable successes exist with all-atom representations of ribosomal subunits or small functional regions.^329,330^ CG MD has explored folding pathways with ribosomal subunits present,^331,332^ and coarser Go-like models with a single bead per amino acid can also be utilized to study these systems^333^ if practitioners accept sacrificing structural and topological detail. Steered molecular dynamics (SMD) accesses otherwise rare events^334^ by adding force along reaction coordinates, determining binding site affinities,^335^ and can therefore be employed to effectively study nascent chains exiting the ribosome tunnel.^333^ A detailed 2023 review by Bock et al.^328^ explores how simulations complement experiments for complex ribosomal biochemistry.

## 6 Implications of Membrane Protein Folding in Disease

The integral relation between protein structure and function has long been understood.^336^ When a protein misfolds, it can cause significant disruptions in cellular function,^337,338^ by reducing the amount of functional and folded protein available in the cell. The balance between folded and misfolded populations can be very thermodynamically delicate; for example, the *in vivo* efficiency for the folding of the human wild-type PMP22 protein is reportedly around 20%.^339–341^ This is sufficient for the appropriate population levels for healthy function in humans. Because the folded state is only slightly favored over misfolded or degraded states, even a small destabilizing mutation with just a single hydrogen bond, ion pair, or hydrophobic interaction disrupted can make a functionally inactive mutant the dominant state within the ensemble of accessible protein states. Such mutations promote a decrease in folding efficiency, which can also be brought about by other factors including changes in the folding environment.^342^ Any decrease in folding efficiency can then have serious consequences – if too few proteins fold correctly, cellular function becomes impaired, leading to disease. Pathology arises when the level of functional, folded protein is below the level needed for normal health, or if the accumulated population of misfolded proteins becomes toxic.^1^

A typical human genome encodes 10,000–12,000 coding variants relative to the reference sequence.^1^ Because each person carries two alleles per gene, these variants imply that roughly half of the protein-coding loci in a typical genome are non-wild-type (i.e., carry at least one alternate coding allele).^343^ By comparison, there are more than 110,000,000 human gene sequence variations validated in the gnomAD (Genome Aggregation Database) across the entire human genome, including noncoding and rare variants.^344,345^ This leads to significant differences in pharmacological profiles among individuals and a very diverse range of pathology in protein-related diseases. While virtually all disease-linked genetic variations that are commonplace are known and catalogued, novel rarer genetic mutations are consistently being discovered as genetic sequencing becomes more high-throughput and commonplace.^345^ The next breakthrough will be the ability to predict which mutations will be benign or pathogenic,^346^ and recent machine learning approaches are seeing a degree of success as the amount of data they have to work with is increasing.^347,348^

Mutations that promote the misfolding of membrane proteins are known contributors to many human diseases.^1^ Furthermore, it is hypothesized that the cumulative effect of misfolded proteins throughout the proteostatic network can be the main driver of pathology rather than a single protein defect.^349–352^ Disease can also arise from somatic mutations that arise spontaneously in a single cell.^353^ The prevalence of diseases directly caused by membrane protein misfolding can be estimated through searching the UNIPROT database,^354^ which reveals that around 20% of the proteome are disease-linked human proteins. A sizable portion of these proteins have at least one TM segment, and there is strong evidence that misfolded proteins are the strongest promoters of disease. Many disease-linked membrane proteins are mistrafficked within the cell,^355–357^ and while misfolding is not the only mechanism for mistrafficking, it is a major cause due to the intimate link between folding and trafficking along the ER to the plasma membrane. Furthermore, pathogenic mutations in disease-linked membrane proteins tend not to cluster within a functional site or domain but are positioned to destabilize the native fold by perturbing the integral interactions between the TM helices,^358–363^ which with current limited experimental methodologies, we fail to completely understand. For example, there are 96 sites in the human vasopressin V2 receptor that are known disease mutations, and 80 of them are located in the TM helices,^364^ demonstrating the importance in weakening the interactions between these helices. It has been observed that mutations that introduce charged residues into TM domains are common among pathogenic MP mutations,^365^ and previous work has suggested that a large proportion of the mutations reduce the proteins’ conformational stability, increasing cellular mistrafficking.^339,366^ Similar observations were also made for water-soluble proteins.^367–371^ The intended membrane environment for MPs is a stabilizing one^372^ that allows the protein to retain its structure, ensuring that the protein can be folded sufficiently to reach this environment to perform its function, even if less efficiently.

An approach to ‘rescue’ misfolded proteins and possibly prevent disease is the use of pharmacological chaperones, or pharmacoperones.^373^ They were discovered in the early 2000s^374–377^ when an antagonist of the vasopressin receptor was observed to “act like a pharmalogical chaperone which promoted receptor processing through their specific binding activity”. Proteins utilize chaperones to assist in their assembly or folding but the chaperone itself is not present in the final protein structure.^378^ They have two primary functions; (1) preventing aggregation of misfolded or unfolded proteins, and (2) assisting the folding of misfolded proteins.^373^ Pharmacoperones operate similarly, binding strongly to a single target protein, to stabilize native conformations, or facilitating the folding of non-native intermediates into their native structure.^378–381^ Crucially, pharmacoperones have minimal inhibitory activity for their target protein,^382,383^ and they do not interfere with the degradation of other misfolded proteins in the ER.^384,385^ Their specific binding is the hallmark that distinguishes pharmacoperones from other small molecules that can be used for binding and folding proteins.^383^ Pharmacoperones enter cells and act as a scaffold for misfolded proteins retained at the ER to adopt a more stable and functional conformation. Their utility is most pronounced for membrane proteins that structurally most closely resemble wild type proteins – some mutated proteins may have their essential TM helix interactions weakened to the extent that they cannot be energetically refolded into a functional form.^386^ There has been some success in the synthesis of pharmacoperone drugs that can modulate the function of proteins trafficked to their intended destination, despite the protein mutations affecting crucial domains related to function,^379^ and *in vitro* and *in vivo* examples of pharmacoperones in correcting the folding of misfolded proteins.^383,386–389^ These compounds are seen as a way to enhance and support the ER quality control system to ensure that the population of misfolded proteins does not reach toxic levels, behaving similarly to the endogenous chaperone molecules but being much more targeted.^390,391^ Protein targets of pharmacoperones include enzymes, transporters, receptors, regulators, and hormones, and a significant portion of their targets are localized in the plasma membrane.^383^ Given their highly targeted approach, recent *in silico* developments predicting protein structures with a paired ligand or inhibitor that takes advantage of deep learning diffusion methods^290,392^ yield an opportunity for pharmacoperones to be designed more rapidly without the need for any preliminary laboratory work before validation. This opens an avenue to address concerns that much of the proteome is not intrinsically refoldable on physiologically relevant time scales^393^ and making the development of pharmacoperones more high-throughput. Pharmacoperones have been more extensively applied to proteins with a loss-of-function mutation^394^ than those with a gain-of-function mutation, which reflects that such mutations appear to be much more common, as loss-of-function is typically brought about by the mutation-induced enhancement of protein misfolding.^368,370,395,396^

## 7 Conclusions and Outlook

Understanding how membrane proteins fold cotranslationally remains a central challenge in biochemistry. This question is important not only for fundamental biology but also because membrane proteins are highly represented among therapeutic targets.^104,397^ Structural studies of cotranslational membrane protein biogenesis are undergoing a clear transition, shifting from low-resolution biochemical experiments and snapshots of isolated complexes toward high-resolution structural and kinetically resolved ribosome–translocon assemblies examined within native membranes and *in situ*. Although this progress is substantial, it has also highlighted several fundamental questions that remain unresolved (Figure 1).

First, when do individual transmembrane helices acquire stable secondary structure during synthesis? While topology and early insertion events can now be followed with reasonable confidence, the precise timing of helix formation within the bilayer is not well-defined. Second, do helices insert and assemble sequentially as they emerge from the ribosome, or do they engage cooperatively to form higher order structure? Closely related is the question of when a stable fold first appears – does this occur in the ribosome exit tunnel, when interacting laterally on the membrane, or once the polypeptide is spanning the bilayer while in the translocon or the membrane itself. Third, how do membrane composition and integration apparatus such as the translocon impose provisional topologies or folds that may later be remodelled in the plasma membrane in eukaryotes? Finally, we ask - how do kinetic factors shape these outcomes? This final question is particularly challenging to address, given that direct observation of membrane protein folding in real-time during translation and within native bilayers is still not possible. Time-resolved SEIRAS holds much promise in this regard but is technically challenging and requires further optimization.

At present, most experimental approaches provide information on topology and early insertion. Biochemical mapping strategies have defined membrane orientation and solvent accessibility for many substrates, but these methods often rely on stalled ribosome–nascent chain complexes or thermodynamically relaxed end-states (Figure 7). Biochemical experiments, along with Cryo-EM have significantly expanded our understanding of the molecular players involved in cotranslational insertion and have been used to hypothesize rules of membrane engagement (Figure 3, Figure 4). However, without quantitative measurements of elongation, insertion, and folding kinetics, these structural snapshots provide limited mechanistic insight into the order and timing of structural acquisition and a decisive mechanistic role of integration apparatus. Moreover, these structures represent isolated states and therefore cannot readily capture or be directly linked to the transient conformational ensembles that form during the dynamic process of cotranslational folding.

Recently, SEIRAS (Figure 8) and advanced fluorescence-based approaches, when coupled with IVTT (Figure 6), have enabled the real-time monitoring of helix formation and membrane insertion under defined conditions. These approaches make it possible to relate structural acquisition directly to the translation elongation rates and membrane composition. Although cell-free systems lack aspects of cellular crowding and chaperone networks, their experimental control provides an essential platform for dissecting the kinetic hierarchy that underpins membrane-protein folding.

Several emerging techniques could help address these outstanding questions about cotranslational membrane protein folding. SMFS approaches have been adapted to study folding directly on stalled RNCs, where the ribosome and nascent chain are tethered in an optical tweezer setup to apply force and monitor folding transitions.^22–25,398,399^ Although this approach has so far been applied only to soluble proteins, future work will determine whether it can be extended to membrane protein RNCs (Section 4.2) to explore kinetic and thermodynamic parameters of helix packing within membranes on ribosomes. HDX-MS is another powerful structural biology technique that reports on protein dynamics by measuring the exchange of backbone amide hydrogens with deuterons in solution.^400,401^ When combined with designed RNC constructs stalled at defined translation lengths, HDX-MS can map folding intermediates along the cotranslational pathway^402^ and has been used to study soluble proteins and their interactions with ribosomal proteins and chaperones.^14,403–406^ Again, this technique has not yet been applied to membrane proteins, largely because of technical challenges. Advances in Cryo-EM, including single-particle analysis and *in situ* cryoelectron tomography, are also expanding the structural investigation of membrane proteins and ribosome-associated complexes, but they are currently limited by intrinsic conformational heterogeneity.^407,408^ Continued improvements in these techniques, together with the development of time-resolved methodologies,^286^ are likely to provide new opportunities to directly observe cotranslational membrane protein folding.

The next phase of the field must therefore move beyond descriptive topology mapping and end point structures toward a quantitative kinetic framework of cotranslational folding in native membranes. By combining real-time biophysical measurements with state-resolved structural approaches, it should become possible to define how translation dynamics, membrane composition, and integration machinery coordinate to produce correctly folded membrane proteins.

## Abbreviations

aa: Amino acid
ABC: ATP-binding cassette
AE: Anion exchanger
AFM: Atomic force microscopy
AMS: 4-Acetamido-4′-maleimidylstilbene-2,2′-disulfonic acid
bR: Bacteriorhodopsin
BAM: β-barrel assembly machinery
BOS: Back of Sec61 complex
CD: Circular dichroism
CFTR: Cystic fibrosis transmembrane conductance regulator
CG: Coarse-grained
CHAPS: 3-((3-Cholamidopropyl)­dimethylammonio)-1-propanesulfonate
CHAPSO: 3-((3-Cholamidopropyl)­dimethylammonio)-2-hydroxy-1-propanesulfonate
ClC: Chloride-conducting ion channel
CL: Cardiolipin
Cryo-EM: Cryogenic electron microscopy
DAG: Diacylglycerol
DDM: *n*-Dodecyl-β-D-maltoside
DFT: Density functional theory
DHPC: 1,2-Dihexanoyl-*sn*-glycero-3-phosphocholine
DIBMA: Di-isobutylene maleic acid
DMPC: 1,2-Dimyristoyl-*sn*-glycero-3-phosphocholine
DMPG: 1,2-Dimyristoyl-*sn*-glycero-3-phosphoglycerol
DOPE: 1,2-Dioleoyl-*sn*-glycero-3-phosphoethanolamine
DOPC: 1,2-Dioleoyl-*sn*-glycero-3-phosphocholine
DOPG: 1,2-Dioleoyl-*sn*-glycero-3-phosphoglycerol
DPPC: 1,2-Dipalmitoyl-*sn*-glycero-3-phosphocholine
DSC: Differential scanning calorimetry
DSPC: 1,2-Distearoyl-*sn*-glycero-3-phosphocholine
EMC: Endoplasmic reticulum membrane protein complex
ER: Endoplasmic reticulum
FPA: Force-profile analysis
FRET: Förster resonance energy transfer
FT-IR: Fourier-transform infrared spectroscopy
GEL: GET- and EMC-like complex
GET: Guided entry of tail-anchored proteins
GPCR: G protein-coupled receptor
HDX-MS: Hydrogen–deuterium exchange mass spectrometry
HTL: Holo-translocon
IRES: Internal ribosome entry site
ITC: Isothermal titration calorimetry
IVTT: *In vitro* transcription/translation
Lep: Leader peptidase
MD: Molecular dynamics
MFS: Major facilitator superfamily
MP/MPs: Membrane protein(s)
MPT: Multipass translocon
MSP: Membrane scaffold protein
MT: Magnetic tweezers
NAC: Nascent polypeptide-associated complex
NBD: Nucleotide-binding domain
NLP: Nanolipoprotein particle
NMR: Nuclear magnetic resonance
NTA: Nitrilotriacetic acid
OST: Oligosaccharyltransferase
OT: Optical tweezers
PAT: Protein associated with the translocon
PC: Phosphatidylcholine
PE: Phosphatidylethanolamine
PG: Phosphatidylglycerol
PR: Proteorhodopsin
PS: Phosphatidylserine
PTS: Phosphotransferase system
PURE: Protein synthesis using recombinant elements
RAMP: Ribosome-associated membrane protein
RNC/RNCs: Ribosome–nascent chain complex­(es)
RRL: Rabbit reticulocyte lysate
SDS: Sodium dodecyl sulfate
SEIRAS: Surface-enhanced infrared absorption spectroscopy
SERT: Serotonin transporter
SLB: Supported lipid bilayer
SM: Sphingomyelin
SMFS: Single-molecule force spectroscopy
SMR: Small multidrug resistance
SMD: Steered molecular dynamics
SPC: Signal peptidase complex
SR: Signal recognition particle receptor
SRP: Signal recognition particle
TA: Tail-anchored
TEM: Transmission electron microscopy
TM/TMs: Transmembrane helix/helices
TMD/TMDs: Transmembrane domain(s)
TRAM: Translocating chain-associating membrane protein
TRAP: Translocon-associated protein complex
TXTL: Transcription–translation
UBA: Ubiquitin-associated (domain)
WGE: Wheat germ extract
